# Predictive value of systemic inflammatory indices for new-onset atrial fibrillation after acute myocardial infarction: an interpretable machine learning study

**DOI:** 10.1080/07853890.2026.2759368

**Published:** 2026-11-11

**Authors:** Xiangzhu Meng, Yuhang Wen, Ge Zhan, Yi Xia, Xiangying Wang, Chengming Ma, Yunlong Xia, Xiaolei Yang, Lianjun Gao

**Affiliations:** a Department of Cardiology, Institute of Cardiovascular Diseases, the First Affiliated Hospital of Dalian Medical University, Dalian, China; b Department of Rehabilitation, Jiangxi Province Hospital of Integrated Chinese and Western Medicine, Nanchang, China

**Keywords:** Systemic inflammatory indices, acute myocardial infarction, new-onset atrial fibrillation, machine learning

## Abstract

**Background:**

New-onset atrial fibrillation (NOAF) after acute myocardial infarction (AMI) is associated with adverse outcomes. Inflammation contributes to atrial fibrillation (AF) onset and progression. We developed an interpretable machine learning (ML) framework integrating complete blood count (CBC)-derived inflammatory indices with routine clinical parameters for NOAF risk assessment.

**Methods:**

We retrospectively analyzed 3,077 AMI patients without prior AF (2018–2023). Multivariable logistic regression, restricted cubic splines (RCS), and threshold analyses evaluated six inflammatory indices: neutrophil-to-lymphocyte ratio (NLR), platelet-to-lymphocyte ratio (PLR), systemic immune-inflammation index (SII), monocyte-to-lymphocyte ratio (MLR), systemic inflammation response index (SIRI), and aggregate index of systemic inflammation (AISI). Boruta and LASSO selections were intersected. Eight ML models were evaluated, with Shapley Additive Explanations (SHAP) for interpretation.

**Results:**

NOAF incidence was 7.73%. Six indices were associated with NOAF in separate adjusted models (all *p* < 0.05). Estimated change points were approximately 5.5 (NLR), 1217 (SII), 2.9 (SIRI), and 430 (AISI). The support vector machine (SVM) achieved the highest mean training out-of-fold (OOF) area under the precision–recall curve (PR-AUC; 0.357) and a held-out test PR-AUC of 0.407. Mean absolute SHAP values were highest for left atrial diameter (LAD), age, and B-type natriuretic peptide (BNP); SIRI ranked first among inflammatory features. Fixed-feature ablation showed modest PR-AUC and Brier-score improvements, uncertain ROC-AUC gains, and no consistent net benefit on decision-curve analysis.

**Conclusions:**

CBC-derived inflammatory indices were associated with NOAF, but their incremental predictive value beyond routine clinical variables remains uncertain. Given moderate predictive performance and no external validation, multicenter validation is needed before clinical implementation.

## Graphical Abstract

**Figure:**
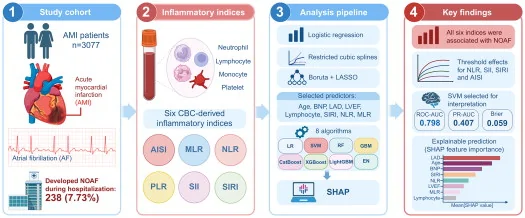

## Introduction

As the most common sustained cardiac arrhythmia in clinical practice, atrial fibrillation (AF) has a continuously rising global prevalence, particularly among the elderly, placing a substantial burden on public health [1]. New-onset atrial fibrillation (NOAF) is the most prevalent supraventricular arrhythmia complicating acute myocardial infarction (AMI), with a reported incidence ranging from 6% to 21% [2]. Its occurrence is a reliable predictor of adverse outcomes, significantly elevating the risks of heart failure, thromboembolism, and stroke, and ultimately compromising long-term survival [3,4]. Managing this embolic risk creates a major therapeutic dilemma: adding anticoagulant therapy to guideline-directed antiplatelet regimens substantially increases bleeding complications [5,6]. Existing clinical risk scores and post-infarction models frequently rely on pre-existing chronic comorbidities or require complex calculations, potentially limiting their utility for early risk assessment of NOAF during the acute phase of AMI. There is a need for accessible biomarkers that capture the acute physiological stress and inflammatory burden at admission to facilitate early risk stratification.

The pathogenesis of NOAF following AMI is complex and multifactorial, involving ischemia-reperfusion injury, myocardial fibrosis, atrial electrical remodeling, hemodynamic disturbances, and neurohumoral dysregulation [7–9]. Growing evidence indicates that inflammation and oxidative stress are pivotal drivers of this arrhythmogenic substrate [10]. Classic inflammatory markers like CRP, IL-6, and TNF-α are involved in this pathological process [11], but some, particularly IL-6 and TNF-α, are less routinely available in acute clinical settings. By contrast, systemic inflammatory indices derived from complete blood counts—calculated from routinely measured platelets, monocytes, lymphocytes, and neutrophils—may reflect aspects of systemic inflammation and oxidative stress. Composite biomarkers such as the neutrophil-to-lymphocyte ratio (NLR), platelet-to-lymphocyte ratio (PLR), monocyte-to-lymphocyte ratio (MLR), systemic immune-inflammation index (SII), systemic inflammation response index (SIRI), and aggregate index of systemic inflammation (AISI) have shown promise in reflecting the interplay of distinct immune pathways. These indices are clinically accessible and have demonstrated prognostic value in cardiovascular diseases, with some showing superior predictive performance compared with individual blood cell counts [12,13].

These six biomarkers can capture overlapping dimensions of the immune-inflammatory response. Their inherent multicollinearity may complicate the evaluation of individual associations in conventional statistical models. Machine learning (ML) algorithms may capture complex patterns and nonlinear relationships among clinical variables, potentially improving risk prediction [14]. Combined with Shapley Additive Explanations (SHAP), ML models can improve interpretability by visualizing and quantifying how individual features contribute to model predictions. However, the comparative predictive value of these indices for in-hospital NOAF after AMI remains unclear. We therefore aimed to evaluate these indices and develop and internally validate an interpretable ML model.

## Materials and methods

### Study design and population

This retrospective observational study was performed at the First Affiliated Hospital of Dalian Medical University. We screened consecutive patients diagnosed with AMI between January 2018 and January 2023. AMI was diagnosed according to the guidelines issued by the European Society of Cardiology (ESC), the American Heart Association (AHA), and the American College of Cardiology (ACC) [15,16]. Exclusion criteria were as follows: (1) moderate to severe renal or hepatic impairment; (2) thyroid dysfunction or electrolyte disturbances; (3) severe valvular heart disease or congenital heart disease; (4) history of cardiac surgery; (5) absence of coronary angiography (CAG); (6) hematological disorders, malignant tumors, active infections, autoimmune diseases, or recent use of medications affecting white blood cell counts; (7) history of AF or atrial flutter (AFL), or AF/AFL documented at admission; and (8) incomplete clinical data. Patient selection is summarized in Supplementary Figure 1. The study was approved by the Ethics Committee of the First Affiliated Hospital of Dalian Medical University (Approval No: PJ-KS-KY-2026-443) and conducted in accordance with the Declaration of Helsinki. The data were anonymized, and the requirement for written informed consent was waived by the Ethics Committee due to the retrospective nature of the study. Patient data confidentiality was strictly maintained throughout the study. This study followed the REporting of studies Conducted using Observational Routinely-collected health Data (RECORD) guidelines [17]. The formal analysis of these data was initiated on 20 March 2026.

### Definitions

NOAF was defined as AF newly documented during hospitalization in patients without a history of AF or atrial flutter (AFL), confirmed by a standard 12-lead ECG or by an AF episode lasting ≥30 s on continuous ECG or 24-hour Holter monitoring [18]. All patients underwent continuous ECG monitoring for at least 24 h after admission, with longer monitoring when clinically indicated. Rhythm strips from continuous monitoring were reviewed by the treating cardiology team. Daily 12-lead ECG was performed during hospitalization, and additional ECG or 24-hour Holter monitoring was obtained for suspected arrhythmias or other clinical indications. Twelve-lead ECG and 24-hour Holter recordings were interpreted by physicians specializing in electrocardiography. Coronary artery stenosis was defined as ≥50% luminal narrowing in any major coronary artery (left main, left anterior descending, left circumflex, or right coronary artery) on CAG [19]. Killip class was defined as follows: Class I, no clinical evidence of heart failure; Class II, pulmonary crackles involving <50% of the lung fields; Class III, pulmonary crackles affecting >50% of the lung fields; and Class IV, cardiogenic shock with significant hemodynamic instability [20]. The CHA_2_DS_2_-VASc score was calculated using standard criteria: 1 point for congestive heart failure, hypertension, age 65–74 years, diabetes mellitus, vascular disease (previous myocardial infarction or peripheral artery disease), and female sex; 2 points for age ≥75 years or prior stroke/transient ischemic attack (TIA) [21].

### Evaluation of systemic inflammatory indices

In this study, six systemic inflammatory indices derived from the complete blood count (CBC) were evaluated to assess their associations with NOAF. Laboratory parameters were obtained from blood samples collected on admission before CAG. The indices were calculated as follows:

- NLR = neutrophil count/lymphocyte count
- PLR = platelet count/lymphocyte count
- SII = (neutrophil count × platelet count)/lymphocyte count
- MLR = monocyte count/lymphocyte count
- SIRI = (neutrophil count × monocyte count)/lymphocyte count
- AISI = (neutrophil count × monocyte count × platelet count)/lymphocyte count

### Data collection

We extracted clinical data from the electronic medical records of all enrolled patients. Variables were categorized as follows: (1) demographic and clinical characteristics: age, sex, body mass index (BMI), admission systolic and diastolic blood pressure, current smoking and alcohol consumption status; (2) laboratory parameters: white blood cell (WBC) count, neutrophil count, lymphocyte count, monocyte count, platelet count, red blood cell (RBC) count, hemoglobin, high-density lipoprotein cholesterol (HDL-C), low-density lipoprotein cholesterol (LDL-C), triglycerides (TG), total cholesterol (TC), D-dimer, uric acid, B-type natriuretic peptide (BNP), creatinine, alanine aminotransferase (ALT), aspartate aminotransferase (AST), high-sensitivity cardiac troponin I (hs-cTnI), glucose, and serum albumin; (3) echocardiographic parameters: left atrial diameter (LAD), left ventricular end-diastolic diameter (LVEDD), and left ventricular ejection fraction (LVEF), measured by transthoracic echocardiography within 24 h after admission; (4) comorbidities: history of hypertension, diabetes mellitus (DM), and prior stroke; (5) clinical severity and procedural details: Killip class, AMI type (STEMI or NSTEMI), and coronary angiographic findings; and (6) medications administered during the index AMI hospitalization, including aspirin, P2Y12 receptor inhibitors, statins, β-blockers, angiotensin-converting enzyme inhibitors (ACEIs) and angiotensin receptor blockers (ARBs), and diuretics. Among patients who developed NOAF, baseline laboratory and echocardiographic measurements preceded the first documented NOAF episode.

### Statistical analysis

Continuous variables with normal distribution were presented as mean ± SD and compared using Student’s t-test, while those with non-normal distribution were expressed as median (interquartile range [IQR]) and analyzed using the Mann–Whitney U test. Categorical variables were summarized as counts and percentages (n, %), with group differences compared using the χ^2^ test or Fisher’s exact test. Patients with incomplete clinical data were excluded, and variable-specific missingness was summarized in Supplementary Table 1. For conventional multivariable regression analyses, collinearity was assessed and covariates with a variance inflation factor (VIF) ≥5 were excluded (Supplementary Figure 2).

To assess associations between systemic inflammatory indices and NOAF, each index was modeled separately as a continuous variable (per 1-SD increase) and by quartiles (Q1–Q4) using four progressively adjusted logistic regression models. Model 1 was unadjusted. Model 2 adjusted for age, sex, BMI, and smoking status. Model 3 further adjusted for hypertension, DM, stroke, and Killip class ≥ II. Model 4 further adjusted for BNP, hs-cTnI, D-dimer, AST, uric acid, creatinine, TG, HDL-C, LDL-C, LAD, and LVEF. Results were expressed as odds ratios (ORs) with 95% confidence intervals (CIs). In a sensitivity analysis addressing differential rhythm surveillance, NOAF was restricted to events first documented during the initial 24-hour continuous ECG monitoring period, using the same adjustment models. Nonlinear associations were assessed using restricted cubic splines (RCS) with three knots at the 10th, 50th, and 90th percentiles of each inflammatory index, with the median as the reference; overall and nonlinear associations were tested using Wald χ^2^ tests. Knot locations and reference values are provided in Supplementary Table 2. When significant nonlinearity was detected, exploratory two-piecewise logistic regression was used to estimate change points. Model fit was compared using likelihood ratio tests, and 95% CIs for change points were estimated from 1,000 bootstrap resamples. Analyses were conducted using SPSS Statistics 31.0 (IBM, USA), R 4.5.2 (R Foundation for Statistical Computing, Austria), and Zstats 1.0 (www.zstats.net). A two-tailed *p* < 0.05 was considered statistically significant.

### Machine learning analysis

After patients with incomplete clinical data were excluded, the dataset was randomly split into training and test cohorts at a 7:3 ratio through stratified sampling. Eight ML classifiers were evaluated: Gradient Boosting Machine (GBM), Logistic Regression (LR), Categorical Boosting (CatBoost), Extreme Gradient Boosting (XGBoost), Random Forest (RF), Elastic Net (EN), Light Gradient Boosting Machine (LightGBM), and Support Vector Machine (SVM). Within the training cohort, models were evaluated using two repeats of stratified 10-fold outer cross-validation (CV), with 3-fold inner CV for hyperparameter tuning based on the area under the precision–recall curve (PR-AUC). Identical fold partitions were preserved across all algorithms. All preprocessing, feature selection, and resampling were confined to training partitions to prevent data leakage. Continuous predictors were standardized using training-partition estimates. Feature selection was based on the intersection of Boruta and least absolute shrinkage and selection operator (LASSO) regression. The LASSO penalty was determined using 10-fold stratified CV and the lambda.1se criterion, with lambda.min used if no variables were selected. If the intersection was empty, the LASSO-selected variables were retained.

Class imbalance was addressed using the Synthetic Minority Oversampling Technique (SMOTE) for continuous-only predictors or SMOTE-NC for mixed continuous and categorical predictors, followed by Random Under-Sampling (RUS). Resampling produced a nearly 1:1 class ratio in the training data, while all validation folds and the held-out test set remained unresampled [22]. The algorithm with the highest mean training out-of-fold (OOF) PR-AUC across the two repeats was selected. For final model fitting, features were selected from the complete training cohort, and hyperparameters were optimized using 3-fold CV. Hyperparameter search spaces and final selected settings are provided in Supplementary Table 3. Platt scaling, fitted to training-set OOF probabilities averaged across the two repeats, was applied to recalibrate predicted probabilities in the held-out internal test set.

In accordance with the TRIPOD+AI reporting guidelines [23], final model performance on the held-out internal test set was evaluated using discrimination metrics (ROC-AUC, PR-AUC, sensitivity, specificity, F1-score, and G-mean), overall prediction error (Brier score), calibration (intercept, slope, and calibration curves), and decision-curve analysis (DCA) across threshold probabilities of 2%–20%. Classification thresholds were determined *via* the Youden index based on calibrated training-set OOF predictions. Paired differences in ROC-AUC and PR-AUC between SVM and the other seven algorithms were assessed using 1,000 outcome-stratified paired bootstrap resamples, reporting 95% bootstrap percentile confidence intervals and Holm-adjusted P values separately for ROC-AUC and PR-AUC.

We performed a post hoc fixed-feature ablation analysis to assess the incremental contribution of the selected inflammatory features after feature selection on the complete training cohort. Using the selected algorithm, three models based on clinical and routine complete blood count (CBC) variables, inflammatory indices, or their combination were compared under identical resampling and tuning procedures. Feature selection was not repeated within the ablation CV folds. Paired changes in ROC-AUC, PR-AUC, Brier score, calibration intercept and slope, and DCA net benefit were assessed in the held-out internal test set using 1,000 stratified bootstrap resamples.

The selected model was interpreted using Shapley Additive Explanations (SHAP), computed using kernelshap and visualized using shapviz in R. The SHAP background comprised 200 randomly selected observations from the non-resampled training set; 403 outcome-stratified test observations were used for interpretation. Global feature importance was quantified using mean absolute SHAP values. Feature-ranking stability was assessed across 200 bootstrap refits, with the selected predictors and hyperparameters held fixed. R package versions are reported in Supplementary Table 4. The global random seed for ML development was 2024.

## Results

### Baseline clinical characteristics

A total of 5,012 patients with AMI admitted between 2018 and 2023 were initially screened, of whom 3,077 were enrolled based on predefined inclusion and exclusion criteria. Table 1 summarizes the baseline characteristics stratified by NOAF occurrence. Patients who developed NOAF were older, had higher CHA_2_DS_2_-VASc scores, longer hospital stays, and a higher prevalence of hypertension, prior stroke, and Killip class ≥ II than those without NOAF. The NOAF group had a lower proportion of men and current smokers (*p* < 0.05 for both). Laboratory and echocardiographic data showed that patients with NOAF had higher levels of all six systemic inflammatory indices, WBC, neutrophil counts, cardiac and renal biomarkers (BNP, hs-cTnI, creatinine, uric acid), AST, D-dimer, and glucose, as well as larger LVEDD and LAD. In the NOAF group, LVEF, RBC counts, lymphocyte counts, platelet counts, albumin, and lipid levels (TC, TG, LDL-C) were significantly lower (*p* < 0.05). The use of β-blockers and diuretics was more common in patients with NOAF. No significant differences were observed between the two groups in BMI, blood pressure, diabetes history, monocyte counts, culprit lesion distribution, AMI type, or use of other routine medications (aspirin, statins, or ACEI/ARB) (*p* > 0.05).

**Table 1.** Baseline characteristics of the study population.

Variables	Total (3077)	NOAF (238)	Non-NOAF (2839)	*P*-value
**Basic characteristics**				
Age (years)	62.05 ± 11.78	69.40 ± 10.12	61.44 ± 11.70	<0.001
Gender (male), n (%)	2349 (76.34%)	167 (70.17%)	2182 (76.86%)	0.024
Current smoking, n (%)	1511 (49.11%)	94 (39.50%)	1417 (49.91%)	0.003
Current drinking, n (%)	1009 (32.79%)	76 (31.93%)	933 (32.86%)	0.824
SBP, mmHg	132.02 ± 23.65	129.26 ± 27.84	132.25 ± 23.26	0.062
DBP, mmHg	78.63 ± 13.29	78.17 ± 15.94	78.67 ± 13.05	0.581
BMI, kg/m^2^	24.59 ± 3.48	24.50 ± 3.78	24.60 ± 3.46	0.671
Killip class ≥ II, n (%)	1842 (59.86%)	161 (67.65%)	1681 (59.21%)	0.013
CHA_2_DS_2_-VASc score	3.00 (2.00, 4.00)	4.00 (3.00, 5.00)	3.00 (2.00, 4.00)	<0.001
Length of hospitalization, days	5.00 (4.00, 7.00)	6.00 (5.00, 7.00)	5.00 (4.00, 7.00)	<0.001
**Medical history**				
Diabetes mellitus, n (%)	975 (31.69%)	74 (31.09%)	901 (31.74%)	0.894
Hypertension, n (%)	1700 (55.25%)	158 (66.39%)	1542 (54.31%)	<0.001
Stroke, n (%)	225 (7.31%)	37 (15.55%)	188 (6.62%)	<0.001
**Laboratory tests**				
WBC,10^9^/L	8.70 ± 2.80	9.23 ± 3.33	8.66 ± 2.74	0.002
RBC, 10^12^/L	4.55 ± 0.53	4.48 ± 0.56	4.56 ± 0.53	0.029
Lymphocyte, 10^9^/L	1.81 ± 0.72	1.52 ± 0.64	1.83 ± 0.72	<0.001
Monocyte, 10^9^/L	0.57 ± 0.24	0.60 ± 0.27	0.57 ± 0.24	0.062
Neutrophil, 10^9^/L	6.19 ± 2.61	7.00 ± 3.09	6.13 ± 2.55	<0.001
Platelet, 10^9^/L	214.34 ± 56.08	205.43 ± 55.45	215.09 ± 56.08	0.011
Hemoglobin, g/L	139.50 ± 16.16	137.88 ± 16.88	139.64 ± 16.09	0.107
D-dimer, mg/L	0.25 (0.16, 0.45)	0.41 (0.23, 0.82)	0.24 (0.15, 0.43)	<0.001
AST, U/L	46.00 (24.00, 124.00)	61.00 (29.25, 172.25)	46.00 (24.00, 120.00)	<0.001
ALT, U/L	30.00 (19.00, 47.00)	32.00 (21.00, 55.00)	30.00 (19.00, 47.00)	0.067
BNP, pg/mL	122.60 (53.30, 274.99)	336.90 (144.90, 607.81)	114.63 (49.81, 250.46)	<0.001
hs-cTnI, ug/L	7.75 (1.10, 42.39)	15.95 (1.87, 77.54)	7.36 (1.05, 41.54)	<0.001
TC, mmol/L	4.82 ± 1.14	4.50 ± 1.06	4.85 ± 1.14	<0.001
TG, mmol/L	1.85 ± 1.10	1.47 ± 0.92	1.88 ± 1.10	<0.001
LDL-C, mmol/L	2.77 ± 0.83	2.56 ± 0.78	2.79 ± 0.83	<0.001
HDL-C, mmol/L	1.05 ± 0.26	1.05 ± 0.24	1.05 ± 0.26	0.996
Creatinine, umol/L	72.78 ± 18.21	79.34 ± 21.93	72.23 ± 17.75	<0.001
Glucose, mmol/L	6.94 ± 3.05	7.35 ± 3.76	6.91 ± 2.98	0.030
Albumin, g/L	39.24 ± 3.34	38.17 ± 3.41	39.33 ± 3.32	<0.001
Uric acid, umol/L	354.34 ± 94.06	368.34 ± 105.19	353.16 ± 92.99	0.017
NLR	3.22 (2.26, 4.90)	4.52 (2.92, 6.97)	3.15 (2.22, 4.74)	<0.001
PLR	120.22 (92.72, 162.96)	137.82 (103.15, 189.69)	118.78 (92.01, 160.97)	<0.001
SII	679.25 (453.77, 1046.73)	898.15 (578.98, 1335.34)	664.53 (447.73, 1021.05)	<0.001
MLR	0.31 (0.23, 0.42)	0.38 (0.29, 0.52)	0.30 (0.23, 0.41)	<0.001
SIRI	1.71 (1.08, 2.92)	2.57 (1.50, 4.04)	1.67 (1.05, 2.83)	<0.001
AISI	357.96 (216.75, 627.98)	466.92 (281.05, 823.18)	348.87 (212.39, 610.80)	<0.001
**Medication**				
Aspirin, n (%)	3031 (98.51%)	231 (97.06%)	2800 (98.63%)	0.102
P2Y12 receptor inhibitor, n (%)	3074 (99.90%)	237 (99.58%)	2837 (99.93%)	0.562
Statins, n (%)	3057 (99.35%)	235 (98.74%)	2822 (99.40%)	0.424
β-blockers, n (%)	2474 (80.40%)	207 (86.97%)	2267 (79.85%)	0.010
ACEI/ARB, n (%)	2040 (66.30%)	154 (64.71%)	1886 (66.43%)	0.639
Diuretic, n (%)	1157 (37.60%)	164 (68.91%)	993 (34.98%)	<0.001
**Echocardiographic data**				
LAD, mm	37.64 ± 4.01	41.75 ± 5.53	37.30 ± 3.65	<0.001
LVEF, %	55.00 (46.00, 58.00)	47.00 (39.00, 55.00)	55.00 (47.00, 58.00)	<0.001
LVEDD, mm	48.73 ± 4.89	50.19 ± 5.38	48.61 ± 4.82	<0.001
**Culprit lesion**				
Left main artery, n (%)	99 (3.22%)	10 (4.20%)	89 (3.13%)	0.570
Left circumflex artery, n (%)	528 (17.16%)	50 (21.01%)	478 (16.84%)	0.235
Left anterior descending artery, n (%)	1318 (42.83%)	95 (39.92%)	1223 (43.08%)	0.475
Right coronary artery, n (%)	1020 (33.15%)	76 (31.93%)	944 (33.25%)	0.737
**Type of AMI**				
STEMI, n (%)	1233 (40.07%)	92 (38.66%)	1141 (40.19%)	0.693
NSTEMI, n (%)	1844 (59.93%)	146 (61.34%)	1698 (59.81%)	

Data are expressed as the means ± SDs, median (interquartile ranges), or numbers (percentages).

Abbreviations: NOAF, new-onset atrial fibrillation; SBP, systolic blood pressure; DBP, diastolic blood pressure; BMI, body mass index; WBC, white blood cells; RBC, red blood cells; ALT, alanine aminotransferase; AST, aspartate aminotransferase; TC, total cholesterol; TG, triglyceride; LDL-C, low-density lipoprotein cholesterol; HDL-C, high-density lipoprotein cholesterol; NLR, neutrophil-to-lymphocyte ratio; PLR, platelet-to-lymphocyte ratio; SII, systemic immune-inflammation index; MLR, monocyte-to-lymphocyte ratio; SIRI, systemic inflammation response index; AISI, aggregate index of systemic inflammation; ACEI, angiotensin-converting enzyme inhibitors; ARB, angiotensin receptor blockers; LVEF, left ventricular ejection fraction; LAD, left atrial diameter; LVEDD, left ventricular end-diastolic dimension; BNP, B-type natriuretic peptide; hs-cTnI, high-sensitivity cardiac troponin I; STEMI, ST-segment elevation myocardial infarction; NSTEMI, non-ST-segment elevation myocardial infarction.

### Rhythm monitoring and NOAF ascertainment

Detailed information on rhythm monitoring and NOAF ascertainment is provided in Supplementary Table 5. All patients received at least 24 h of continuous ECG monitoring after admission. Monitoring duration, the proportion monitored beyond 24 h, and 24-h Holter use did not differ significantly between groups (all *p* > 0.05). Among the 238 patients with NOAF, AF was first documented by continuous ECG monitoring in 143 (60.1%), 12-lead ECG in 65 (27.3%), and 24-h Holter monitoring in 30 (12.6%). The median time from admission to first documented NOAF was 26.6 h (IQR, 12.0–49.0 h), with 115 cases (48.3%) first documented within 24 h of admission.

### Association between inflammatory biomarkers and NOAF

Supplementary Figure 3 shows that the proportion of patients with NOAF increased progressively across ascending quartiles for all inflammatory markers (all *P* for trend < 0.001). Logistic regression analysis (Table 2) showed that these positive associations persisted after adjustment for prespecified clinical covariates. In the fully adjusted Model 4, each 1-SD increase in SIRI was associated with 34% higher odds of NOAF (OR = 1.34, 95% CI: 1.17–1.53, *p* < 0.001). Similar associations per 1-SD rise were observed for NLR and MLR (OR = 1.30 for both, 95% CI: 1.13–1.49, *p* < 0.001), AISI (OR = 1.28, 95% CI: 1.12–1.46, *p* < 0.001), SII (OR = 1.26, 95% CI: 1.10–1.44, *p* < 0.001), and PLR (OR = 1.15, 95% CI: 1.01–1.31, *p* = 0.038). Compared with Q1, patients in the highest quartile (Q4) of SIRI, NLR, and AISI had higher odds of NOAF, with ORs of 2.61 (95% CI: 1.61–4.33), 2.67 (95% CI: 1.65–4.55), and 2.46 (95% CI: 1.53–4.03), res­pectively (all *p* < 0.001). Significant associations were also observed for SII, MLR, and PLR (Q4 *vs.* Q1), with ORs of 2.08 (95% CI: 1.31–3.37, *p* = 0.002), 1.98 (95% CI: 1.23–3.25, *p* = 0.006), and 1.63 (95% CI: 1.05–2.57, *p* = 0.033), respectively. After full adjustment, all six biomarkers showed positive quartile trends (all *P* for trend < 0.05). In the sensitivity analysis restricted to NOAF first documented within the initial 24-h continuous ECG monitoring window, the associations of all six inflammatory indices remained directionally consistent and statistically significant after full multivariable adjustment (Supplementary Table 6).

**Table 2.** Association between complete blood count (CBC)-derived inflammatory biomarkers and NOAF by logistic regression.

	Model 1		Model 2		Model 3		Model 4	
Exposure	OR (95% CI)	*P*	OR (95% CI)	*P*	OR (95% CI)	*P*	OR (95% CI)	*P*
NLR (per SD increase)	1.48 (1.33, 1.64)	<0.001	1.47 (1.31, 1.64)	<0.001	1.47 (1.31, 1.64)	<0.001	1.30 (1.13, 1.49)	<0.001
NLR quartiles								
Q1	1(Ref)		1(Ref)		1(Ref)		1(Ref)	
Q2	1.38 (0.84, 2.29)	0.208	1.43 (0.87, 2.39)	0.162	1.45 (0.88, 2.42)	0.152	1.21 (0.71, 2.07)	0.491
Q3	2.41 (1.54, 3.85)	<0.001	2.43 (1.54, 3.91)	<0.001	2.42 (1.54, 3.90)	<0.001	1.87 (1.15, 3.11)	0.014
Q4	4.33 (2.86, 6.77)	<0.001	4.15 (2.71, 6.53)	<0.001	4.09 (2.67, 6.45)	<0.001	2.67 (1.65, 4.55)	<0.001
*P* for trend	1.68 (1.47, 1.91)	<0.001	1.64 (1.44, 1.87)	<0.001	1.62 (1.42, 1.86)	<0.001	1.42 (1.22, 1.66)	<0.001
PLR (per SD increase)	1.34 (1.19, 1.50)	<0.001	1.26 (1.12, 1.42)	<0.001	1.25 (1.11, 1.41)	<0.001	1.15 (1.01, 1.31)	0.038
PLR quartiles								
Q1	1(Ref)		1(Ref)		1(Ref)		1(Ref)	
Q2	1.30 (0.85, 2.02)	0.228	1.24 (0.80, 1.93)	0.343	1.19 (0.76, 1.85)	0.450	1.13 (0.70, 1.83)	0.631
Q3	1.64 (1.09, 2.50)	0.019	1.42 (0.94, 2.18)	0.103	1.37 (0.90, 2.11)	0.143	1.36 (0.86, 2.17)	0.197
Q4	2.39 (1.63, 3.57)	<0.001	2.02 (1.36, 3.04)	<0.001	1.93 (1.29, 2.91)	0.001	1.63 (1.05, 2.57)	0.033
*P* for trend	1.34 (1.18, 1.51)	<0.001	1.26 (1.11, 1.43)	<0.001	1.25 (1.10, 1.42)	<0.001	1.18 (1.03, 1.36)	0.018
SII (per SD increase)	1.35 (1.21, 1.49)	<0.001	1.40 (1.25, 1.56)	<0.001	1.40 (1.25, 1.56)	<0.001	1.26 (1.10, 1.44)	<0.001
SII quartiles								
Q1	1(Ref)		1(Ref)		1(Ref)		1(Ref)	
Q2	1.28 (0.81, 2.05)	0.291	1.37 (0.86, 2.20)	0.187	1.34 (0.84, 2.15)	0.225	1.01 (0.62, 1.68)	0.958
Q3	2.07 (1.36, 3.19)	<0.001	2.14 (1.40, 3.34)	<0.001	2.08 (1.35, 3.24)	0.001	1.64 (1.03, 2.65)	0.040
Q4	3.01 (2.03, 4.58)	<0.001	3.33 (2.22, 5.10)	<0.001	3.22 (2.15, 4.95)	<0.001	2.08 (1.31, 3.37)	0.002
*P* for trend	1.47 (1.30, 1.67)	<0.001	1.51 (1.33, 1.72)	<0.001	1.50 (1.32, 1.71)	<0.001	1.32 (1.14, 1.53)	<0.001
MLR (per SD increase)	1.50 (1.35, 1.67)	<0.001	1.44 (1.28, 1.62)	<0.001	1.43 (1.27, 1.60)	<0.001	1.30 (1.13, 1.49)	<0.001
MLR quartiles								
Q1	1(Ref)		1(Ref)		1(Ref)		1(Ref)	
Q2	1.34 (0.84, 2.18)	0.227	1.37 (0.84, 2.23)	0.206	1.38 (0.85, 2.25)	0.197	1.30 (0.78, 2.20)	0.321
Q3	2.35 (1.53, 3.68)	<0.001	2.32 (1.50, 3.66)	<0.001	2.32 (1.49, 3.67)	<0.001	1.82 (1.13, 3.00)	0.016
Q4	3.44 (2.29, 5.30)	<0.001	3.11 (2.04, 4.86)	<0.001	2.99 (1.96, 4.69)	<0.001	1.98 (1.23, 3.25)	0.006
*P* for trend	1.54 (1.36, 1.75)	<0.001	1.48 (1.30, 1.69)	<0.001	1.45 (1.28, 1.66)	<0.001	1.25 (1.08, 1.46)	0.003
SIRI (per SD increase)	1.42 (1.28, 1.57)	<0.001	1.48 (1.33, 1.65)	<0.001	1.47 (1.32, 1.64)	<0.001	1.34 (1.17, 1.53)	<0.001
SIRI quartiles								
Q1	1(Ref)		1(Ref)		1(Ref)		1(Ref)	
Q2	1.50 (0.95, 2.40)	0.084	1.61 (1.01, 2.59)	0.047	1.61 (1.01, 2.60)	0.047	1.42 (0.86, 2.37)	0.172
Q3	1.92 (1.24, 3.01)	0.004	1.98 (1.27, 3.14)	0.003	2.01 (1.29, 3.20)	0.002	1.77 (1.08, 2.93)	0.024
Q4	3.45 (2.31, 5.28)	<0.001	4.02 (2.66, 6.24)	<0.001	3.90 (2.57, 6.06)	<0.001	2.61 (1.61, 4.33)	<0.001
*P* for trend	1.51 (1.33, 1.71)	<0.001	1.58 (1.38, 1.80)	<0.001	1.56 (1.37, 1.78)	<0.001	1.37 (1.17, 1.59)	<0.001
AISI (per SD increase)	1.30 (1.17, 1.44)	<0.001	1.40 (1.25, 1.57)	<0.001	1.39 (1.24, 1.55)	<0.001	1.28 (1.12, 1.46)	<0.001
AISI quartiles								
Q1	1(Ref)		1(Ref)		1(Ref)		1(Ref)	
Q2	1.62 (1.04, 2.56)	0.035	1.67 (1.06, 2.65)	0.028	1.64 (1.04, 2.61)	0.034	1.38 (0.85, 2.28)	0.200
Q3	1.99 (1.30, 3.11)	0.002	2.08 (1.35, 3.27)	0.001	2.06 (1.34, 3.24)	0.001	1.71 (1.06, 2.78)	0.030
Q4	2.96 (1.98, 4.53)	<0.001	3.61 (2.39, 5.59)	<0.001	3.47 (2.29, 5.38)	<0.001	2.46 (1.53, 4.03)	<0.001
*P* for trend	1.41 (1.24, 1.59)	<0.001	1.51 (1.33, 1.72)	<0.001	1.49 (1.31, 1.70)	<0.001	1.34 (1.16, 1.56)	<0.001

Model 1: adjusted for none.

Model 2: adjusted for age, gender, BMI, and current smoking.

Model 3: adjusted for age, gender, BMI, current smoking, hypertension, DM, Stroke, Killip class ≥ II.

Model 4: adjusted for age, gender, BMI, current smoking, hypertension, DM, Stroke, Killip class ≥ II, BNP, hs-cTnI, D-dimer, AST, Uric acid, creatinine, TG, HDL-C, LDL-C, LAD, and LVEF.

Abbreviations: NOAF, new-onset atrial fibrillation; BMI, body mass index; DM, diabetes mellitus; AST, aspartate aminotransferase; TG, triglyceride; HDL-C, high-density lipoprotein cholesterol; hs-cTnI, high-sensitivity cardiac troponin I; LDL-C, low-density lipoprotein cholesterol; LVEF, left ventricular ejection fraction; LAD, left atrial diameter; NLR, neutrophil-to-lymphocyte ratio; PLR, platelet-to-lymphocyte ratio; SII, systemic immune-inflammation index; MLR, monocyte-to-lymphocyte ratio; SIRI, systemic inflammation response index; AISI, aggregate index of systemic inflammation; Ref, reference; SD, standard deviation; OR, odds ratio; CI, confidence interval.

After adjustment for Model 4 covariates, RCS analysis revealed nonlinear associations of NLR, SII, SIRI, and AISI with NOAF (all P for nonlinearity < 0.05; Figure 1). Exploratory two-piecewise logistic regression estimated change points at approximately 5.5 for NLR, 1217 for SII, 2.9 for SIRI, and 430 for AISI, with bootstrap-derived 95% CIs reported in Supplementary Table 7. The estimated associations were stronger below their respective change points, whereas none of the upper-segment associations reached statistical significance. These data-derived change points should not be interpreted as validated clinical cut-offs.

**Figure 1.**
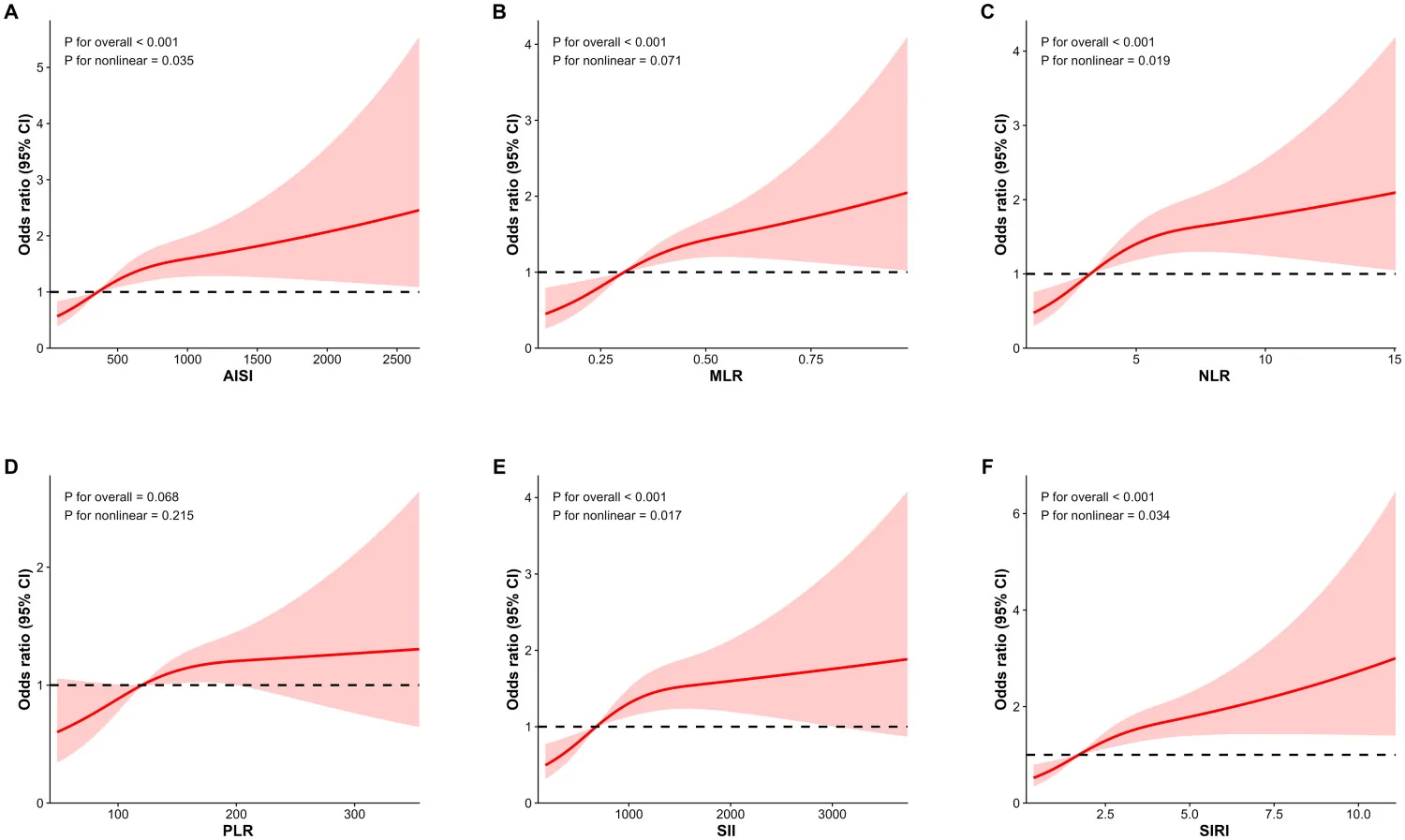
Dose–response associations between six inflammatory indices and NOAF based on restricted cubic spline (RCS) logistic regression, adjusted for Model 4. (A) AISI, (B) MLR, (C) NLR, (D) PLR, (E) SII, and (F) SIRI. Abbreviations: NOAF, new-onset atrial fibrillation; NLR, neutrophil-to-lymphocyte ratio; PLR, platelet-to-lymphocyte ratio; SII, systemic immune-inflammation index; MLR, monocyte-to-lymphocyte ratio; SIRI, systemic inflammation response index; AISI, aggregate index of systemic inflammation.

### Subgroup analysis

Subgroup analyses by sex, age group, smoking status, alcohol consumption, diabetes, hypertension, and stroke showed no significant interactions (all *P* for interaction > 0.05; Supplementary Figure 4), and the associations between these inflammatory biomarkers and NOAF remained consistent across subgroups.

### Feature selection for ML

Baseline characteristics were balanced between the training (*n* = 2,155) and test (*n* = 922) sets, with all absolute standardized mean differences (SMDs) below 0.1 (Supplementary Table 8). Feature selection for the final model was performed on the complete training cohort. LASSO regression with 10-fold stratified cross-validation retained eight predictors using the lambda.1se criterion (Figure 2A,B), while Boruta identified 23 relevant features (Figure 2C). The intersection comprised LAD, age, BNP, MLR, NLR, LVEF, SIRI, and lymphocyte count, which were retained for subsequent ML and SHAP analyses (Figure 2D).

**Figure 2.**
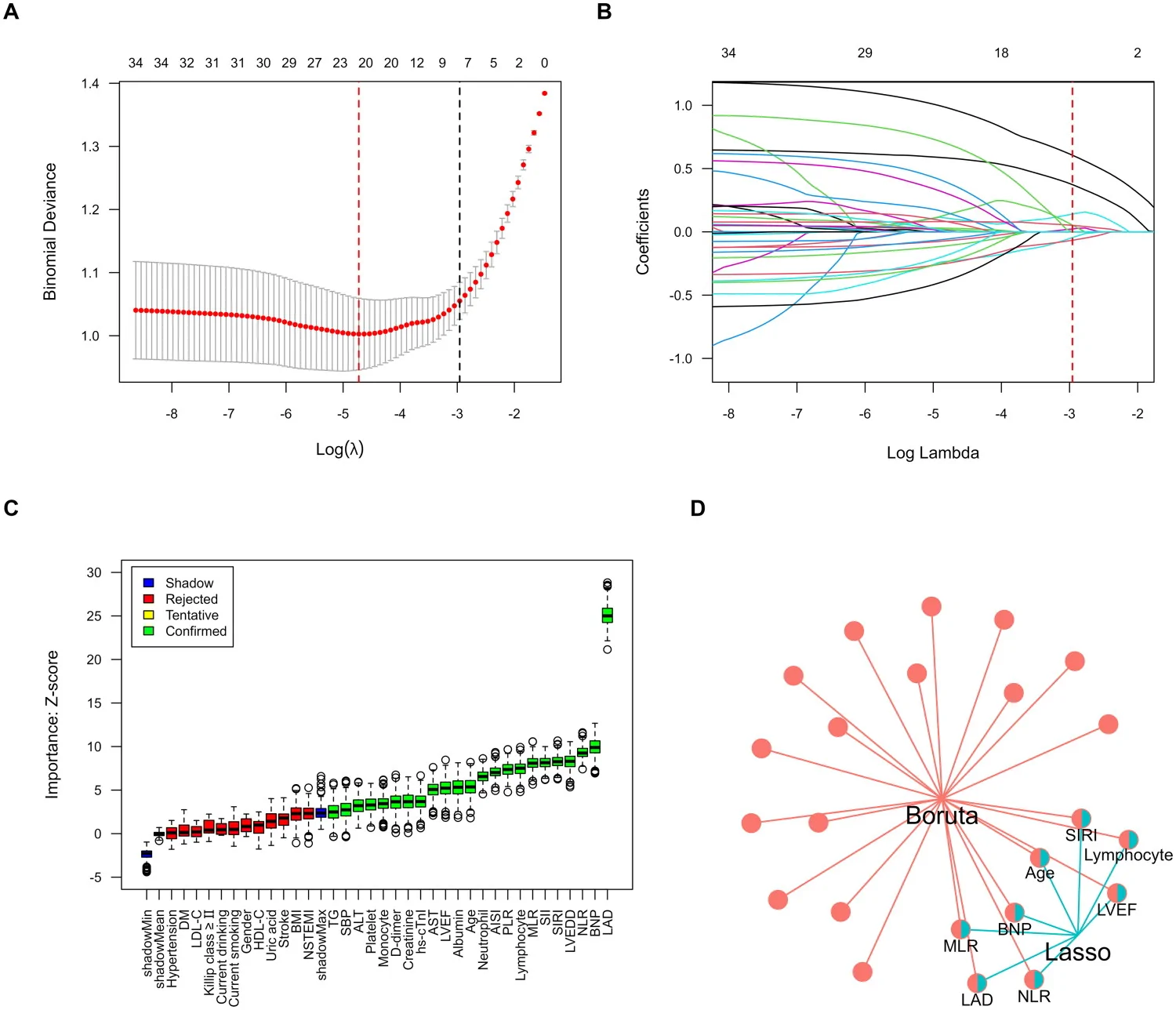
Feature selection for NOAF predictors *via* LASSO and Boruta. (A) LASSO selection by cross-validated binomial deviance using the λ.1se criterion; (B) LASSO coefficient paths against log(λ); (C) Boruta results: green, yellow, and red for confirmed, tentative, and rejected features; (D) Network of overlapping features from both methods. Abbreviations: NOAF, new-onset atrial fibrillation.

### Construction and evaluation of ML models

Eight ML models were evaluated using two repeats of stratified 10-fold outer cross-validation with 3-fold inner tuning, followed by held-out internal testing (Figure 3; Table 3). SVM achieved the highest mean training out-of-fold (OOF) PR-AUC (0.357, SD 0.001), followed by LR (0.348, SD 0.008), and was selected for subsequent SHAP analysis. In the test set, SVM achieved the highest PR-AUC (0.407), with a ROC-AUC of 0.798, a PR-AUC lift of 5.284, and a Brier score of 0.059, whereas CatBoost achieved the highest ROC-AUC (0.801). After OOF-based Platt scaling, the SVM calibration intercept and slope were 0.098 and 0.923, respectively. DCA showed no consistent net benefit advantage for SVM across thresholds of 2%–20%. Paired bootstrap comparisons showed a higher PR-AUC for SVM than RF (ΔPR-AUC = 0.151, 95% CI 0.074–0.219; Holm-adjusted *p* = 0.007), but no other PR-AUC or ROC-AUC differences were statistically significant after Holm adjustment (Supplementary Table 9).

**Figure 3.**
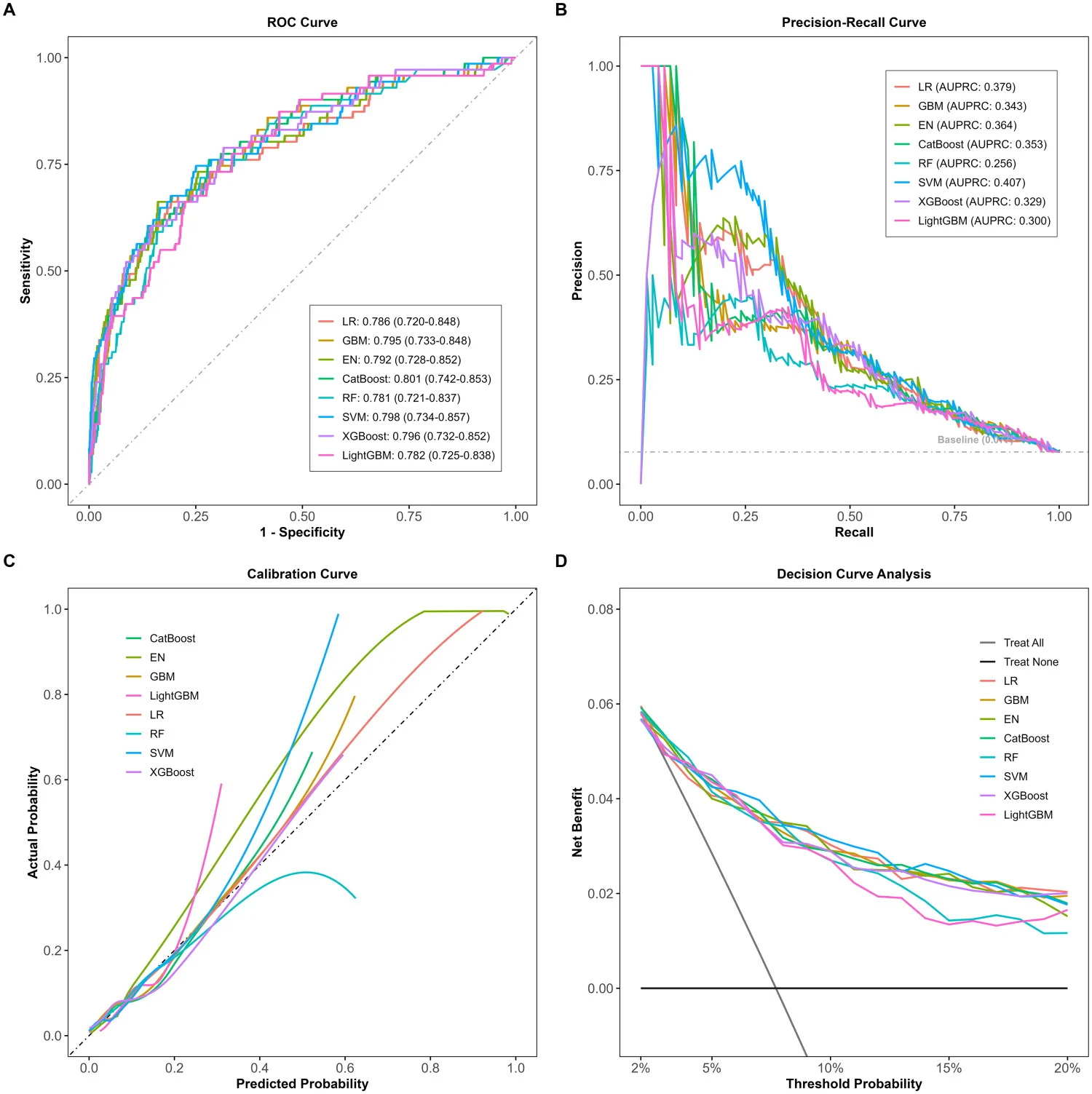
Predictive performance of eight machine learning models for NOAF. (A) ROC curves; (B) Precision-recall curves; (C) Calibration curves; (D) DCA for each model. Abbreviations: NOAF, new-onset atrial fibrillation; ROC, receiver operating characteristic; AUPRC, area under the precision-recall curve; LR, logistic regression; SVM, support vector machine; GBM, gradient boosting machine; RF, random forest; XGBoost, extreme gradient boosting; LightGBM, light gradient boosting machine; CatBoost, Categorical Boosting; EN, Elastic Net; DCA, decision curve analysis.

**Table 3.** Performance of eight ML models in the held-out internal test set.

Model	ROC-AUC (95% CI)	PR-AUC (95% CI)	PR-AUC lift (95% CI)	Accuracy (95% CI)	Sensitivity (95% CI)	Specificity (95% CI)	G-mean (95% CI)	F1-score (95% CI)	Brier score
LR	0.786 (0.720, 0.848)	0.379 (0.272, 0.498)	4.923 (3.530, 6.472)	0.781 (0.753, 0.807)	0.676 (0.555, 0.782)	0.790 (0.761, 0.817)	0.731 (0.665, 0.792)	0.322 (0.271, 0.374)	0.059
XGBoost	0.796 (0.732, 0.852)	0.329 (0.239, 0.443)	4.276 (3.102, 5.759)	0.675 (0.643, 0.705)	0.789 (0.676, 0.877)	0.665 (0.632, 0.697)	0.724 (0.673, 0.771)	0.272 (0.237, 0.306)	0.061
LightGBM	0.782 (0.725, 0.838)	0.300 (0.209, 0.399)	3.902 (2.716, 5.184)	0.771 (0.744, 0.796)	0.606 (0.493, 0.718)	0.785 (0.757, 0.811)	0.689 (0.618, 0.751)	0.290 (0.237, 0.341)	0.064
SVM	0.798 (0.734, 0.857)	0.407 (0.296, 0.521)	5.284 (3.843, 6.767)	0.819 (0.792, 0.843)	0.634 (0.511, 0.745)	0.834 (0.808, 0.859)	0.727 (0.656, 0.791)	0.350 (0.292, 0.410)	0.059
GBM	0.795(0.733, 0.848)	0.343 (0.241, 0.444)	4.459 (3.126, 5.763)	0.747 (0.718, 0.775)	0.690 (0.569, 0.795)	0.752 (0.722, 0.781)	0.720 (0.657, 0.776)	0.296 (0.251, 0.340)	0.061
RF	0.781 (0.721, 0.837)	0.256 (0.187, 0.360)	3.327 (2.433, 4.681)	0.623 (0.590, 0.654)	0.789 (0.676, 0.877)	0.609 (0.575, 0.642)	0.693 (0.642, 0.736)	0.243 (0.212, 0.272)	0.064
CatBoost	0.801 (0.742, 0.853)	0.353 (0.250, 0.455)	4.586 (3.242, 5.909)	0.729 (0.699, 0.757)	0.704 (0.584, 0.807)	0.731 (0.700, 0.760)	0.717 (0.657, 0.770)	0.286 (0.242, 0.327)	0.061
EN	0.792 (0.728, 0.852)	0.364 (0.256, 0.479)	4.728 (3.327, 6.218)	0.773 (0.745, 0.800)	0.676 (0.555, 0.782)	0.781 (0.752, 0.809)	0.727 (0.662, 0.787)	0.315 (0.265, 0.367)	0.062

Abbreviations: ML, machine learning; LR, logistic regression; XGBoost, extreme gradient boosting; SVM, support vector machine; GBM, gradient boosting machine; RF, random forest; LightGBM, light gradient boosting machine; CatBoost, categorical boosting; EN, elastic net; CI, confidence interval; ROC-AUC, area under the receiver operating characteristic curve; PR-AUC, area under the precision–recall curve; F1-score, harmonic mean of precision and recall; G-mean, geometric mean of sensitivity and specificity.

In the post hoc fixed-feature ablation analysis, the inflammation-only model achieved a test ROC-AUC of 0.645 and a PR-AUC of 0.127. The combined model showed a numerically higher test PR-AUC (0.407 *vs.* 0.354) and a lower Brier score (0.0587 *vs.* 0.0605) than the clinical/CBC model. Paired bootstrap analysis showed a modest reduction in Brier score (Δ = −0.00185, 95% CI: −0.00383 to −0.00005) and an increase in PR-AUC (Δ = 0.0532, 95% CI: 0.0014–0.1152), whereas the improvement in ROC-AUC remained uncertain. DCA showed no consistent additional net benefit across threshold probabilities of 2%–20% (Supplementary Table 10).

### Model interpretation

SHAP analysis was used to interpret the fitted SVM model. LAD and age had the largest mean absolute SHAP values, followed by BNP and SIRI (Figure 4A). The beeswarm plot summarized the direction and distribution of feature contributions across individuals (Figure 4B). At the individual level, waterfall and force plots illustrated how feature contributions shifted predictions from a background-based baseline probability of approximately 9.0% (Supplementary Figure 5). For the patient with NOAF, LAD, BNP, and age provided the largest positive contributions, partially offset by negative contributions from SIRI, MLR, and NLR, resulting in a predicted probability above baseline. For the patient without NOAF, negative contributions from LAD, NLR, SIRI, MLR, and lymphocyte count outweighed the positive contributions, principally from age and BNP, resulting in a predicted probability below baseline. Dependence plots showed that higher LAD, age, BNP, SIRI, NLR, and MLR generally corresponded to more positive SHAP contributions, whereas higher LVEF and lymphocyte counts tended to correspond to more negative contributions; these patterns were not uniformly monotonic (Supplementary Figure 6). Across 200 bootstrap refits, LAD and age were ranked among the top three features in 100.0% and 98.5% of refits, respectively. Among the inflammatory features, SIRI most frequently ranked in the top three (35.0%). The mean pairwise Spearman correlation between feature rankings across refits was 0.63.

**Figure 4.**
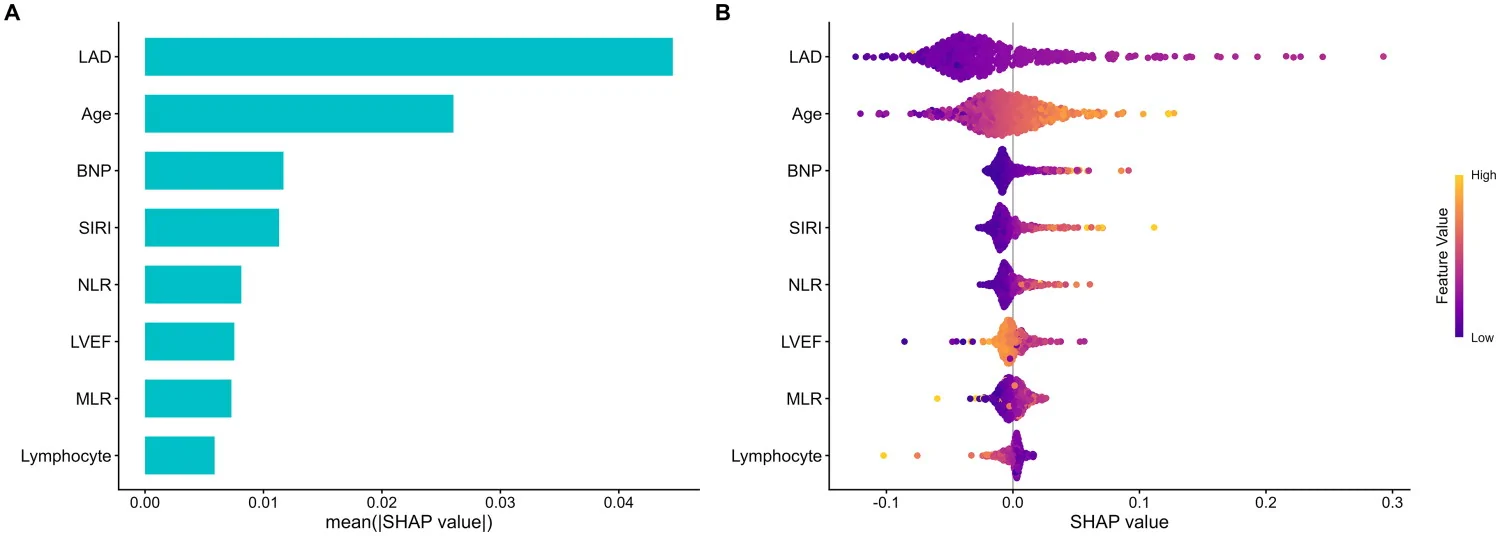
Global interpretation of the SVM model using SHAP analysis. (A) Bar plot of feature importance based on mean absolute SHAP values; (B) Beeswarm plot showing the distribution of SHAP values across features. Abbreviations: SVM, support vector machine; SHAP, Shapley Additive Explanations; LVEF, left ventricular ejection fraction; LAD, left atrial diameter; BNP, B-type natriuretic peptide; NLR, neutrophil-to-lymphocyte ratio; MLR, monocyte-to-lymphocyte ratio; SIRI, systemic inflammation response index.

## Discussion

In this large-scale retrospective cohort study comprising 3,077 AMI patients without a history of AF who underwent CAG at our hospital, we evaluated six systemic inflammatory indices derived from complete blood counts (AISI, MLR, NLR, PLR, SII, and SIRI) for predicting NOAF. Elevated levels of all six indices were significantly associated with increased NOAF risk. RCS analyses revealed distinct dose-response trends across these indices, with several exhibiting significant nonlinear associations; subsequent exploratory two-piecewise regression estimated change points. The principal contribution of the present study is the systematic comparative assessment of six routinely available CBC-derived inflammatory indices within a unified interpretable machine-learning framework for in-hospital NOAF after AMI. These findings suggest that routinely available systemic inflammatory indices may provide additional predictive information, although their incremental clinical utility beyond routine clinical variables remains uncertain.

Despite advances in AMI management with modern reperfusion strategies and guideline-directed medical therapies, NOAF remains a common complication linked to poor outcomes. In our cohort, NOAF occurred in 7.73% of patients, aligning with prior findings [2]. Because many NOAF episodes are transient or asymptomatic, they are often missed by routine monitoring, suggesting that this figure likely underestimates the true clinical burden [24]. Furthermore, evidence indicates that post-AMI NOAF is not merely an acute event but a marker of persistent arrhythmogenic risk, as evidenced by high recurrence rates during long-term follow-up [25]. In this context, these accessible inflammatory markers may warrant further evaluation as adjuncts to clinical risk stratification.

In the fitted SVM model, SHAP analysis indicated that LAD had the largest SHAP contribution to the model’s predictions. Left atrial structural remodeling, pathologically characterized by myocardial fibrosis and echocardiographic atrial enlargement, is fundamentally intertwined with AF initiation and progression [26]. Left atrial dilation can impair intra-atrial conduction and promote reentrant circuit formation, thereby contributing to the arrhythmogenic substrate for NOAF [27,28]. This model-based observation is consistent with previous studies reporting LAD as an independent correlate of NOAF [29]. Other studies and clinical guidelines have also reported that left atrial enlargement is associated with recurrent AF after cardioversion or catheter ablation [21,30]. Reduced LVEF and elevated BNP also contributed to the fitted model’s predictions, a pattern consistent with a plausible hemodynamic pathway. Post-infarction left ventricular systolic dysfunction may raise end-diastolic pressure, leading to retrograde elevation of left atrial pressure and acute atrial myocardial stretch [31]. As a sensitive marker of cardiac strain, BNP reflects both acute ischemic injury and increased ventricular wall tension after AMI [32]. When superimposed on pre-existing atrial structural remodeling, acute hemodynamic stretch may shorten atrial action potential duration and contribute to NOAF [31,33,34]. These findings suggest that BNP and LVEF may reflect hemodynamic burden relevant to NOAF risk, although their incremental predictive value was not evaluated separately in this study.

Advanced age, a well-established risk factor for AF, also emerged as a critical predictive feature in our cohort. Aging drives progressive atrial degenerative changes, including interstitial fibrosis and sinus node dysfunction, which collectively compromise atrial compliance and electrical stability [35]. This structural and functional remodeling creates a vulnerable arrhythmogenic substrate, potentially increasing susceptibility to AF during the acute ischemic and inflammatory stress of AMI. Although the proportion of younger patients among AMI hospitalizations has increased in some populations [36], older patients with impaired cardiac function may remain particularly vulnerable to NOAF during AMI [37].

Systemic inflammatory indices, calculated from neutrophils, lymphocytes, monocytes, and platelets, may reflect partially overlapping dimensions of the inflammatory and immune responses. Following ischemic injury, neutrophils rapidly infiltrate the myocardium, releasing proteolytic enzymes, myeloperoxidase, and reactive oxygen species (ROS) that aggravate endothelial damage and accelerate early atrial remodeling [38–40]. Monocytes migrate to the injured tissue and differentiate into macrophages, propagating the inflammatory cascade [41]. These macrophages drive interstitial fibrosis *via* TGF-β signaling, promoting collagen deposition and the formation of an arrhythmogenic substrate [42]. Activated platelets secrete proinflammatory cytokines independent of hemostasis. Increased platelet volume may alter blood viscosity and exacerbate inflammation [43]. These processes may promote microvascular dysfunction and exacerbate myocardial ischemia, heightening NOAF susceptibility [44,45]. Lymphocytes are essential for inflammatory resolution. Post-AMI activation of the hypothalamic-pituitary-adrenal axis increases glucocorticoid secretion and may promote circulating lymphocyte redistribution to the bone marrow [46]. The resulting lymphopenia may reflect post-infarction physiological stress [47], while regulatory T cells may contribute to the resolution of inflammation during myocardial repair [48]. These composite indices may therefore reflect an imbalance between proinflammatory activity and impaired immune regulation, providing a biologically plausible framework for their observed associations with NOAF in patients with AMI. This interpretation is consistent with the contemporary concept of atrial cardiomyopathy, in which inflammation, oxidative stress, and fibrosis contribute to structural and electrical atrial remodeling and thereby create a substrate that promotes AF susceptibility [49].

NLR and SII have been extensively studied as composite inflammatory indices in cardiovascular research. Although all six indices showed adjusted associations with NOAF in separate logistic regression models, joint LASSO–Boruta feature selection retained SIRI, NLR, and MLR, but not PLR, SII, or AISI, among the final eight predictors. In the fitted SVM model, SIRI had the largest mean absolute SHAP value among the retained inflammatory features, followed by NLR and MLR. The nonselection of PLR, SII, and AISI does not negate their adjusted associations with NOAF and may partly reflect overlapping predictive information among correlated indices; however, the reasons cannot be established from feature selection alone. The retained indices are leukocyte-based, whereas the nonselected indices all incorporate platelet counts. Because these indices share constituent blood-cell components, neither feature selection nor SHAP attributions establish independent biological effects or causal relationships, and the underlying mechanisms were not directly assessed in our study.

Several previous studies have examined inflammatory indices in relation to AF, including NOAF after AMI. Pan et al. reported that NLR, together with the stress hyperglycemia ratio, was associated with NOAF after AMI and included these variables in a conventional nomogram [50]. More recently, Yang et al. compared NLR, LMR, PLR, and SIRI in patients with AMI undergoing emergency PCI and incorporated SIRI into a nomogram for in-hospital NOAF prediction [51]. In a different setting, Hou et al. applied multiple machine-learning algorithms and SHAP to predict AF in patients with coronary heart disease using a composite multi-inflammatory index (MII, calculated as NLR × CRP) [52]. These studies either evaluated selected inflammatory indices for NOAF after AMI using conventional regression models or applied machine learning and SHAP to a composite inflammatory index in broader coronary heart disease populations. In this study, we compared six routinely available CBC-derived inflammatory indices in patients with AMI and used feature selection and an explainable machine-learning framework to characterize the contributions of the selected inflammatory features to model predictions alongside routine clinical variables.

Although SVM achieved a test PR-AUC of 0.407, paired comparisons did not establish its superiority over LR. In the post hoc fixed-feature ablation analysis, the inflammation-only model showed limited predictive performance (test PR-AUC = 0.127). Adding the selected inflammatory indices to the clinical/CBC model modestly improved PR-AUC (0.354 to 0.407) and reduced the Brier score (0.0605 to 0.0587), whereas the improvement in ROC-AUC remained uncertain and DCA showed no consistent additional net benefit. The exploratory DCA thresholds of 2%–20% represented hypothetical cutoffs for closer observation or extended ECG monitoring rather than established clinical decision thresholds. Bootstrap analyses indicated greater ranking stability for LAD and age than for the inflammatory features, supporting caution in interpreting individual SHAP rankings.

The model’s moderate predictive performance, lack of external validation, and absence of consistent evidence of incremental clinical net benefit do not yet support direct clinical use. SHAP attribution patterns may, however, inform hypotheses for future studies of risk-based ECG monitoring or clinical assessment strategies; such hypotheses require independent external validation and prospective evaluation. Our findings indicate associations between CBC-derived inflammatory indices and NOAF, but cannot establish a causal role of inflammation in NOAF or determine whether modifying inflammatory pathways would reduce this risk. These indices should be regarded as risk markers rather than therapeutic targets, and therapeutic implications remain hypothesis-generating. SGLT2 inhibitors have been associated with fewer reported AF/AFL events and reduced post-ablation AF recurrence in other clinical settings, possibly through effects on cardiac remodeling and inflammation [53,54]. Their potential role in acute coronary syndrome has also been discussed, but whether they prevent post-AMI NOAF remains unknown [55]. Low-dose colchicine has been investigated for cardiovascular risk reduction after MI [56]. Whether either therapy can prevent post-AMI NOAF, particularly in patients with elevated inflammatory indices, requires prospective evaluation.

Several limitations should be acknowledged. First, the single-center retrospective design and exclusion of patients with incomplete data may introduce selection bias and limit generalizability. Because the model was validated internally only, independent multicenter external validation is needed. Second, although all patients underwent at least 24 h of continuous ECG monitoring (extended when clinically indicated) and received daily 12-lead ECGs, continuous monitoring was not routinely maintained throughout hospitalization; brief or asymptomatic NOAF episodes may therefore have been missed, potentially underestimating NOAF incidence. Third, inflammatory indices were measured only at admission, leaving the potential value of serial measurements unknown. Fourth, inflammatory cytokines (e.g. IL-6 and TNF-α) and monocyte subpopulations were not assessed, limiting mechanistic interpretation. Fifth, the prognostic value of these biomarkers for post-discharge AF recurrence, stroke, and all-cause mortality was not evaluated. Quartile, spline, threshold, and subgroup analyses were exploratory and require independent validation. Sixth, residual confounding remains possible despite adjustment, particularly because medication dose, duration, and adherence data were unavailable; unrecognized sleep apnea and other factors may also contribute. Finally, the limited number of NOAF events, including 71 in the held-out test set, may have reduced the precision and statistical power of subgroup and model-comparison analyses.

## Conclusion

In conclusion, six readily available CBC-derived inflammatory indices (NLR, PLR, SII, MLR, SIRI, and AISI) were associated with in-hospital NOAF after AMI in separate adjusted models. Within the interpretable ML framework, SIRI was the highest-ranked inflammatory feature in the fitted SVM model, and exploratory analyses suggested nonlinear associations with potential change points for NLR, SII, SIRI, and AISI. However, the incremental clinical utility of the selected inflammatory indices beyond routine clinical and CBC variables was not established, and the SVM model showed moderate predictive performance. Independent multicenter external validation and prospective evaluation are needed before clinical implementation.

## Supplementary Material

Supplemental Material

## Data Availability

The data underlying this article are available from the corresponding author on reasonable request.
