# Supplementary material for: Predictive value of systemic inflammatory indices for new-onset atrial fibrillation after acute myocardial infarction: an interpretable machine learning study
Source: Ann Med. 2026 Nov 11;58(1):2759368. doi: 10.1080/07853890.2026.2759368 (PMC13761050; doi:10.1080/07853890.2026.2759368)
Supplement: Supplementary file 1 [file IANN_A_2759368_SM1355.docx]

**Supplementary Materials**

| **Supplementary Table 1.** Summary of missing data. | | |
| --- | --- | --- |
| Variable | Missing, n | Missing, % |
| Creatinine | 105 | 2.81 |
| Uric acid | 116 | 3.11 |
| BNP | 147 | 3.94 |
| D-dimer | 131 | 3.51 |
| Glucose | 145 | 3.89 |
| LAD | 178 | 4.77 |
| LVEF | 178 | 4.77 |
| LVEDD | 178 | 4.77 |
| **Notes:** Missing percentages were calculated among 3,731 patients before complete-case exclusion. Patients could have missing data for more than one variable. | | |
| **Abbreviations:** LVEF, left ventricular ejection fraction; LAD, left atrial diameter; LVEDD, left ventricular end-diastolic diameter; BNP, B-type natriuretic peptide. | | |

| **Supplementary Table 2.** Knot locations and reference values used in the restricted cubic spline analyses. | | | | |
| --- | --- | --- | --- | --- |
| Inflammatory index | Knot 1 | Knot 2 | Knot 3 | Reference value |
| AISI | 141.08 | 357.96 | 1016.11 | 357.96 |
| MLR | 0.18 | 0.31 | 0.56 | 0.31 |
| NLR | 1.67 | 3.22 | 7.67 | 3.22 |
| PLR | 74.19 | 120.22 | 212.32 | 120.22 |
| SII | 322.61 | 679.25 | 1677.21 | 679.25 |
| SIRI | 0.74 | 1.71 | 4.56 | 1.71 |
| **Note:** Restricted cubic spline models were fitted using three knots placed at the 10th, 50th, and 90th percentiles of each inflammatory index and adjusted for all covariates included in Model 4. The median value of each inflammatory index was used as the reference. | | | | |
| **Abbreviations:** NLR, neutrophil-to-lymphocyte ratio; PLR, platelet-to-lymphocyte ratio; SII, systemic immune-inflammation index; MLR, monocyte-to-lymphocyte ratio; SIRI, systemic inflammation response index; AISI, aggregate index of systemic inflammation. | | | | |

| **Supplementary Table 3.** Hyperparameter search spaces and final selected settings for eight machine-learning algorithms. | | |
| --- | --- | --- |
| **Algorithm** | **Hyperparameter search space / fixed settings** | **Final selected settings** |
| LR | Unpenalized binomial logistic regression; no hyperparameter tuning. | Not applicable. |
| GBM | Fixed grid (25 combinations): n.trees = {50, 100, 150, 200, 250}; interaction.depth = {1, 2, 3, 4, 5}. Fixed: shrinkage = 0.1; n.minobsinnode = 10. | n.trees = 100; interaction.depth = 2; shrinkage = 0.1; n.minobsinnode = 10. |
| EN | Fixed grid (105 combinations): alpha = {0, 0.1, 0.25, 0.5, 0.75, 0.9, 1}; lambda included five prespecified values plus a geometric sequence spanning 10^-4 to 1 (15 unique lambda values). | alpha = 0.5; lambda = 0.129155. |
| CatBoost | Fixed grid (25 combinations): depth = {1, 2, 3, 4, 5}; l2_leaf_reg = {1, 2, 3, 4, 5}. Fixed: learning_rate = 0.05; iterations = 100; rsm = 0.9; border_count = 255. | depth = 3; learning_rate = 0.05; iterations = 100; l2_leaf_reg = 3; rsm = 0.9; border_count = 255. |
| RF | Up to 15 combinations: mtry = {1, 2, 3, 4, 5}; ntree = {300, 500, 800}. Only feasible mtry values were evaluated for each training partition. | mtry = 2; ntree = 800. |
| SVM | Radial basis function (RBF) kernel; fixed 49-combination grid: C = 10^a for a = −1, −0.5, 0, 0.5, 1, 1.5, 2; gamma = 10^b for b = −3, −2.5, −2, −1.5, −1, −0.5, 0. External training-partition standardization; internal e1071 scaling disabled. | kernel = radial (RBF); C = 10; gamma = 0.003162. |
| XGBoost | 30 configurations sampled once (seed 2925) from a 108-combination grid: max_depth = {3, 5, 7}; eta = {0.01, 0.05, 0.1}; subsample = {0.8, 1}; colsample_bytree = {0.8, 1}; min_child_weight = {1, 3, 5}. Up to 500 rounds; early-stopping patience = 30. | max_depth = 3; eta = 0.1; subsample = 0.8; colsample_bytree = 1; min_child_weight = 5; nrounds = 49. |
| LightGBM | 30 configurations sampled once (seed 2926) from a 72-combination grid: max_depth = {3, 5, 7}; learning_rate = {0.01, 0.05, 0.1}; bagging_fraction = {0.8, 1}; feature_fraction = {0.8, 1}; min_data_in_leaf = {10, 20}. Fixed: bagging_freq = 1 when bagging_fraction < 1; otherwise 0. Up to 500 rounds; early-stopping patience = 30. | max_depth = 3; learning_rate = 0.01; bagging_fraction = 0.8; feature_fraction = 0.8; min_data_in_leaf = 20; bagging_freq = 1; nrounds = 61. |
| **Notes:** Hyperparameters were selected by maximizing mean PR-AUC using 3-fold inner cross-validation within two repeats of stratified 10-fold outer cross-validation in the training cohort. Final hyperparameters were tuned using the complete training cohort with the same 3-fold procedure. For XGBoost and LightGBM, boosting rounds were set to the rounded median of the best iterations across the three final tuning folds. Feature selection, standardization, and resampling (SMOTE or SMOTE-NC, followed by random undersampling) were restricted to training partitions. Platt scaling was fitted using training-set out-of-fold predictions. The held-out test set was excluded from model development and tuning. Values are rounded for presentation; exact grid values were used in model fitting. Global random seed: 2024. | | |
| **Abbreviations:** LR, logistic regression; GBM, gradient boosting machine; EN, elastic net; RF, random forest; SVM, support vector machine; RBF, radial basis function; NOAF, new‑onset atrial fibrillation; PR‑AUC, area under the precision‑recall curve; SMOTE‑NC, synthetic minority oversampling technique for nominal and continuous features. | | |

| **Supplementary Table 4.** Software and package versions | |
| --- | --- |
| Package | Version |
| R | 4.5.2 |
| readxl | 1.5.0 |
| Boruta | 10.0.0 |
| ranger | 0.18.0 |
| caret | 7.0.1 |
| PRROC | 1.4 |
| pROC | 1.19.0.1 |
| smotefamily | 1.4.0 |
| glmnet | 5.0 |
| gbm | 2.3.1 |
| randomForest | 4.7.1.2 |
| catboost | 1.2.7 |
| e1071 | 1.7.17 |
| xgboost | 3.2.1.1 |
| lightgbm | 4.7.0 |
| openxlsx | 4.2.8.1 |
| ggplot2 | 4.0.3 |
| patchwork | 1.3.2 |
| recipes | 1.3.3 |
| themis | 1.1.0 |
| scales | 1.4.0 |
| kernelshap | 0.9.1 |
| shapviz | 0.10.3 |
| **Note:** R package versions were extracted from the recorded R sessions for the primary eight-model machine-learning analysis and the SHAP analysis (R 4.5.2). Only R packages directly used in these analyses are listed; non-R software is reported separately. | |

| **Supplementary Table 5.** Rhythm monitoring and ascertainment of NOAF during hospitalization. | | | | |
| --- | --- | --- | --- | --- |
| Characteristic | Total (3077) | NOAF (238) | Non-NOAF (2839) | *P*-value |
| **Rhythm-monitoring protocol** |  |  |  |  |
| Continuous ECG monitoring for ≥24 h, n (%) | 3077 (100.0) | 238 (100.0) | 2839 (100.0) | — |
| Duration of continuous ECG monitoring, h, median (IQR) | 24.0 (24.0, 38.5) | 24.0 (24.0, 39.8) | 24.0 (24.0, 38.4) | 0.403 |
| Continuous ECG monitoring extended beyond 24 h, n (%) | 929 (30.2) | 78 (32.8) | 851 (30.0) | 0.407 |
| Routine daily 12-lead ECG during hospitalization, n (%) | 3077 (100.0) | 238 (100.0) | 2839 (100.0) | — |
| 24-h Holter monitoring during hospitalization, n (%) | 455 (14.8) | 41 (17.2) | 414 (14.6) | 0.313 |
| **NOAF ascertainment (NOAF patients only)** |  |  |  |  |
| First documented by continuous ECG monitoring, n (%) | — | 143 (60.1) | — | — |
| First documented by 12-lead ECG, n (%) | — | 65 (27.3) | — | — |
| First documented by 24-h Holter monitoring, n (%) | — | 30 (12.6) | — | — |
| Time from admission to first documented NOAF, h, median (IQR) | — | 26.6 (12.0, 49.0) | — | — |
| First documented NOAF within 24 h after admission, n (%) | — | 115 (48.3) | — | — |
| **Note:** All patients received ≥24 h continuous ECG monitoring after admission, extended if clinically indicated, followed by daily 12-lead ECGs during hospitalization. Additional ECG or 24-h Holter was performed for suspected arrhythmia symptoms or clinical need. First NOAF episodes were identified by three mutually exclusive modalities in 238 patients. | | | | |
| **Abbreviations:** NOAF, new-onset atrial fibrillation; ECG, electrocardiogram; IQR, interquartile range. | | | | |

| **Supplementary Table 6.** Sensitivity analysis of inflammatory markers and early NOAF within a uniform 24-hour continuous ECG monitoring window. | | | | | | | | |
| --- | --- | --- | --- | --- | --- | --- | --- | --- |
|  | Model 1 | | Model 2 | | Model 3 | | Model 4 | |
| Exposure | OR (95% CI) | *P* | OR (95% CI) | *P* | OR (95% CI) | *P* | OR (95% CI) | *P* |
| AISI | 1.33 (1.15, 1.52) | <0.001 | 1.43 (1.23, 1.65) | <0.001 | 1.41 (1.22, 1.63) | <0.001 | 1.34 (1.13, 1.57) | <0.001 |
| MLR | 1.52 (1.31, 1.75) | <0.001 | 1.44 (1.23, 1.68) | <0.001 | 1.43 (1.22, 1.66) | <0.001 | 1.34 (1.13, 1.59) | <0.001 |
| NLR | 1.56 (1.36, 1.79) | <0.001 | 1.54 (1.33, 1.78) | <0.001 | 1.54 (1.33, 1.77) | <0.001 | 1.39 (1.17, 1.64) | <0.001 |
| PLR | 1.42 (1.21, 1.65) | <0.001 | 1.34 (1.13, 1.57) | <0.001 | 1.33 (1.13, 1.56) | <0.001 | 1.25 (1.05, 1.48) | 0.012 |
| SII | 1.43 (1.24, 1.63) | <0.001 | 1.49 (1.28, 1.71) | <0.001 | 1.48 (1.27, 1.70) | <0.001 | 1.37 (1.15, 1.61) | <0.001 |
| SIRI | 1.45 (1.26, 1.64) | <0.001 | 1.50 (1.30, 1.72) | <0.001 | 1.49 (1.29, 1.71) | <0.001 | 1.40 (1.18, 1.64) | <0.001 |
| **Note:** This sensitivity analysis restricted the outcome to NOAF first documented within the initial 24-hour continuous ECG monitoring window, during which all patients were monitored uniformly. Model 1 was unadjusted. Model 2 was adjusted for age, gender, BMI, and current smoking. Model 3 was further adjusted for hypertension, DM, stroke, and Killip class ≥ II. Model 4 was further adjusted for BNP, hs-cTnI, D-dimer, AST, uric acid, creatinine, TG, HDL-C, LDL-C, LAD, and LVEF, in addition to the covariates in Model 3. Odds ratios are presented per 1-SD increase in each inflammatory index. | | | | | | | | |
| **Abbreviations:** NOAF, new-onset atrial fibrillation; BMI, body mass index; DM, diabetes mellitus; AST, aspartate aminotransferase; TG, triglyceride; HDL-C, high-density lipoprotein cholesterol; hs-cTnI, high-sensitivity cardiac troponin I; LDL-C, low-density lipoprotein cholesterol; LVEF, left ventricular ejection fraction; LAD, left atrial diameter; NLR, neutrophil-to-lymphocyte ratio; PLR, platelet-to-lymphocyte ratio; SII, systemic immune-inflammation index; MLR, monocyte-to-lymphocyte ratio; SIRI, systemic inflammation response index; AISI, aggregate index of systemic inflammation; SD, standard deviation; OR, odds ratio; CI, confidence interval. | | | | | | | | |

| **Supplementary Table 7.** Threshold effect analysis of complete blood count (CBC)-derived inflammatory biomarkers and NOAF | | |
| --- | --- | --- |
| Outcome | Estimate (95% CI) | *P*-value |
| **NLR** |  |  |
| Model 1 Fitting model by standard logistic regression | 1.10 (1.05, 1.16) | <0.001 |
| Model 2 Fitting model by two-piecewise logistic regression |  |  |
| Inflection point | 5.5 (3.4, 8.6) |  |
| <5.5 | 1.28 (1.13, 1.46) | <0.001 |
| ≥5.5 | 1.02 (0.94, 1.10) | 0.681 |
| *P* for likelihood ratio test |  | 0.012 |
| **SII (per 100-unit increase)** |  |  |
| Model 1 Fitting model by standard logistic regression | 1.04 (1.02, 1.06) | <0.001 |
| Model 2 Fitting model by two-piecewise logistic regression |  |  |
| Inflection point | 1217 (776, 1522) |  |
| <1217 | 1.11 (1.05, 1.17) | <0.001 |
| ≥1217 | 1.00 (0.97, 1.04) | 0.936 |
| *P* for likelihood ratio test |  | 0.009 |
| **SIRI** |  |  |
| Model 1 Fitting model by standard logistic regression | 1.17 (1.09, 1.25) | <0.001 |
| Model 2 Fitting model by two-piecewise logistic regression |  |  |
| Inflection point | 2.9 (0.8, 4.6) |  |
| <2.9 | 1.46 (1.17, 1.83) | <0.001 |
| ≥2.9 | 1.09 (0.99, 1.20) | 0.092 |
| *P* for likelihood ratio test |  | 0.035 |
| **AISI (per 100-unit increase)** |  |  |
| Model 1 Fitting model by standard logistic regression | 1.06 (1.03, 1.09) | <0.001 |
| Model 2 Fitting model by two-piecewise logistic regression |  |  |
| Inflection point | 430 (153, 709) |  |
| <430 | 1.26 (1.08, 1.48) | 0.004 |
| ≥430 | 1.03 (1.00, 1.07) | 0.075 |
| *P* for likelihood ratio test |  | 0.024 |
| **Notes:** Adjusted for age, gender, BMI, current smoking, hypertension, DM, stroke, Killip class ≥ II, BNP, hs-cTnI, D-dimer, AST, uric acid, creatinine, TG, HDL-C, LDL-C, LAD, and LVEF. Inflection point was estimated using segmented logistic regression and should be interpreted as exploratory; it was reported on the original index scales, with 95% CIs obtained from 1,000 outcome-stratified bootstrap resamples. Adjusted ORs for SII and AISI were expressed per 100-unit increase, with OR CIs derived from the corresponding regression models. | | |
| **Abbreviations:** NOAF, new-onset atrial fibrillation; BMI, body mass index; DM, diabetes mellitus; AST, aspartate aminotransferase; TG, triglyceride; HDL-C, high-density lipoprotein cholesterol; LDL-C, low-density lipoprotein cholesterol; LVEF, left ventricular ejection fraction; LAD, left atrial diameter; NLR, neutrophil-to-lymphocyte ratio; SII, systemic immune-inflammation index; SIRI, systemic inflammation response index; AISI, aggregate index of systemic inflammation. | | |

| **Supplementary Table 8.** Baseline clinical characteristics in training group and test group. | | | | |
| --- | --- | --- | --- | --- |
| Variables | Total (3077) | Training (2155) | Test (922) | SMD |
| **Basic characteristics** |  |  |  |  |
| Age (years) | 62.05 ± 11.78 | 62.13 ± 11.70 | 61.88 ± 11.98 | 0.021 |
| Gender (male), n (%) | 2349 (76.34%) | 1648 (76.47%) | 701 (76.03%) | 0.010 |
| Current smoking, n (%) | 1511 (49.11%) | 1039 (48.21%) | 472 (51.19%) | 0.060 |
| Current drinking, n (%) | 1009 (32.79%) | 698 (32.39%) | 311 (33.73%) | 0.029 |
| SBP, mmHg | 132.02 ± 23.65 | 131.93 ± 23.84 | 132.23 ± 23.22 | 0.013 |
| DBP, mmHg | 78.63 ± 13.29 | 78.72 ± 13.40 | 78.41 ± 13.05 | 0.024 |
| BMI, kg/m^2^ | 24.59 ± 3.48 | 24.58 ± 3.50 | 24.63 ± 3.44 | 0.015 |
| Killip class ≥ Ⅱ, n (%) | 1842 (59.86%) | 1273 (59.07%) | 569 (61.71%) | 0.054 |
| CHA_2_DS_2_-VASc score | 3.00 (2.00, 4.00) | 3.00 (2.00, 4.00) | 3.00 (2.00, 4.00) | 0.023 |
| Length of hospitalization, days | 5.00 (4.00, 7.00) | 5.00 (4.00, 7.00) | 5.00 (4.00, 7.00) | 0.032 |
| **Medical history** |  |  |  |  |
| Diabetes mellitus, n (%) | 975 (31.69%) | 701 (32.53%) | 274 (29.72%) | 0.061 |
| Hypertension, n (%) | 1700 (55.25%) | 1175 (54.52%) | 525 (56.94%) | 0.049 |
| Stroke, n (%) | 225 (7.31%) | 156 (7.24%) | 69 (7.48%) | 0.009 |
| **Laboratory tests** |  |  |  |  |
| WBC, 10^9^/L | 8.70 ± 2.80 | 8.67 ± 2.82 | 8.77 ± 2.75 | 0.035 |
| RBC, 10^12^/L | 4.55 ± 0.53 | 4.56 ± 0.54 | 4.54 ± 0.52 | 0.033 |
| Lymphocyte, 10^9^/L | 1.81 ± 0.72 | 1.80 ±0.71 | 1.84 ± 0.76 | 0.055 |
| Monocyte, 10^9^/L | 0.57 ± 0.24 | 0.57 ± 0.24 | 0.58 ± 0.25 | 0.039 |
| Neutrophil, 10^9^/L | 6.19 ± 2.61 | 6.18 ± 2.64 | 6.23 ± 2.53 | 0.019 |
| Platelet, 10^9^/L | 214.34 ± 56.08 | 213.70 ± 55.18 | 215.84 ± 58.13 | 0.038 |
| Hemoglobin, g/L | 139.50 ± 16.16 | 139.56 ± 16.38 | 139.36 ± 15.64 | 0.013 |
| D-dimer, mg/L | 0.25 (0.16, 0.45) | 0.25 (0.16, 0.44) | 0.26 (0.16, 0.48) | 0.014 |
| AST, U/L | 46.00 (24.00, 124.00) | 45.00 (24.00, 126.00) | 50.00 (25.00, 122.75) | 0.014 |
| ALT, U/L | 30.00 (19.00, 47.00) | 30.00 (19.00, 47.00) | 30.00 (19.00, 47.00) | 0.026 |
| BNP, pg/mL | 122.60 (53.30, 274.99) | 121.66 (52.85, 276.12) | 126.57 (54.30, 271.18) | 0.022 |
| hs-cTnI, ug/L | 7.75 (1.10, 42.39) | 7.36 (1.01, 42.29) | 9.03 (1.30, 43.03) | 0.011 |
| TC, mmol/L | 4.82 ± 1.14 | 4.82 ± 1.13 | 4.83 ± 1.15 | 0.009 |
| TG, mmol/L | 1.85 ± 1.10 | 1.86 ± 1.12 | 1.82 ± 1.04 | 0.037 |
| LDL-C, mmol/L | 2.77 ± 0.83 | 2.77 ± 0.82 | 2.77 ± 0.85 | <0.001 |
| HDL-C, mmol/L | 1.05 ± 0.26 | 1.05 ± 0.25 | 1.06 ± 0.27 | 0.042 |
| Creatinine, umol/L | 72.78 ± 18.21 | 72.57 ± 17.88 | 73.28 ± 18.96 | 0.039 |
| Glucose, mmol/L | 6.94 ± 3.05 | 6.98 ± 3.04 | 6.86 ± 3.05 | 0.039 |
| Albumin, g/L | 39.24 ± 3.34 | 39.23 ± 3.30 | 39.25 ± 3.43 | 0.006 |
| Uric acid, umol/L | 354.34 ± 94.06 | 354.11 ± 94.37 | 354.87 ± 93.38 | 0.008 |
| NLR | 3.22 (2.26, 4.90) | 3.22 (2.27, 4.94) | 3.23 (2.22, 4.82) | 0.010 |
| PLR | 120.22 (92.72, 162.96) | 120.78 (92.58, 163.32) | 118.77 (92.98, 161.72) | 0.024 |
| SII | 679.25 (453.77, 1046.73) | 678.33 (453.36, 1052.97) | 680.97 (459.84, 1031.26) | 0.005 |
| MLR | 0.31 (0.23, 0.42) | 0.31 (0.23, 0.42) | 0.30 (0.23, 0.42) | 0.021 |
| SIRI | 1.71 (1.08, 2.92) | 1.71 (1.06, 2.90) | 1.72 (1.11, 2.96) | 0.007 |
| AISI | 357.96 (216.75, 627.98) | 354.99 (214.69, 632.69) | 362.22 (223.10, 608.44) | 0.003 |
| **Medication** |  |  |  |  |
| Aspirin, n (%) | 3031 (98.51%) | 2121 (98.42%) | 910 (98.70%) | 0.023 |
| P2Y12 receptor inhibitor, n (%) | 3074 (99.90%) | 2153 (99.91%) | 921 (99.89%) | 0.005 |
| Statins, n (%) | 3057 (99.35%) | 2138 (99.21%) | 919 (99.67%) | 0.062 |
| β-blockers, n (%) | 2474 (80.40%) | 1735 (80.51%) | 739 (80.15%) | 0.009 |
| ACEI/ARB, n (%) | 2040 (66.30%) | 1431 (66.40%) | 609 (66.05%) | 0.007 |
| Diuretic, n (%) | 1157 (37.60%) | 799 (37.08%) | 358 (38.83%) | 0.036 |
| **Echocardiographic data** |  |  |  |  |
| LAD, mm | 37.64 ± 4.01 | 37.68 ± 4.02 | 37.55 ± 3.96 | 0.034 |
| LVEF, % | 55.00 (46.00, 58.00) | 55.00 (46.00, 58.00) | 55.00 (47.00, 58.00) | 0.025 |
| LVEDD, mm | 48.73 ± 4.89 | 48.81 ± 4.94 | 48.55 ± 4.75 | 0.053 |
| **Culprit lesion** |  |  |  |  |
| Left main artery, n (%) | 99 (3.22%) | 67 (3.11%) | 32 (3.47%) | 0.020 |
| Left circumflex artery, n (%) | 528 (17.16%) | 366 (16.98%) | 162 (17.57%) | 0.016 |
| Left anterior descending artery, n (%) | 1318 (42.83%) | 915 (42.46%) | 403 (43.71%) | 0.025 |
| Right coronary artery, n (%) | 1020 (33.15%) | 719 (33.36%) | 301 (32.65%) | 0.015 |
| **Type of AMI** |  |  |  |  |
| STEMI, n (%) | 1233 (40.07%) | 857 (39.77%) | 376 (40.78%) | 0.021 |
| NSTEMI, n (%) | 1844 (59.93%) | 1298 (60.23%) | 546 (59.22%) |  |
| **Outcome** |  |  |  |  |
| NOAF, n (%) | 238 (7.73%) | 167 (7.75%) | 71 (7.70%) | 0.002 |
| **Notes:** Data are expressed as the means± SDs, median (interquartile ranges), or numbers (percentages).  **Abbreviations:** NOAF, new-onset atrial fibrillation; SBP, systolic blood pressure; DBP, diastolic blood pressure; BMI, body mass index; WBC, white blood cells; RBC, red blood cells; ALT, alanine aminotransferase; AST, aspartate aminotransferase; TC, total cholesterol; TG, triglyceride; LDL-C, low-density lipoprotein cholesterol; HDL-C, high-density lipoprotein cholesterol; NLR, neutrophil-to-lymphocyte ratio; PLR, platelet-to-lymphocyte ratio; SII, systemic immune-inflammation index; MLR, monocyte-to-lymphocyte ratio; SIRI, systemic inflammation response index; AISI, aggregate index of systemic inflammation; ACEI, angiotensin-converting enzyme inhibitors; ARB, angiotensin receptor blockers; LVEF, left ventricular ejection fraction; LAD, left atrial diameter; LVEDD, left ventricular end-diastolic dimension; BNP, B-type natriuretic peptide; hs-cTnI, high-sensitivity cardiac troponin I; STEMI, ST-segment elevation myocardial infarction; NSTEMI, non-ST-segment elevation myocardial infarction. | | | | |

| **Supplementary Table 9**. Repeated nested cross-validation performance, calibration, and paired model comparisons for eight machine-learning algorithms. | | | | | | |
| --- | --- | --- | --- | --- | --- | --- |
| **Panel A.** Repeated nested cross-validation performance in the training cohort | | | | | | |
| Model | OOF PR-AUC, mean (SD) | | | OOF ROC-AUC, mean (SD) | | |
| LR | 0.3475 (0.0081) | | | 0.8326 (0.0030) | | |
| XGBoost | 0.3254 (0.0067) | | | 0.8174 (0.0059) | | |
| LightGBM | 0.2874 (0.0393) | | | 0.8026 (0.0096) | | |
| SVM | 0.3573 (0.0013) | | | 0.8337 (0.0022) | | |
| GBM | 0.3089 (0.0016) | | | 0.8206 (0.0052) | | |
| RF | 0.2841 (0.0092) | | | 0.8171 (0.0068) | | |
| CatBoost | 0.2859 (0.0264) | | | 0.8143 (0.0106) | | |
| EN | 0.3239 (0.0223) | | | 0.8227 (0.0012) | | |
| **Panel B.** Calibration performance in the held-out internal test set after Platt scaling trained on OOF predictions | | | | | | |
| Model | Brier score (95% CI) | | Calibration intercept (95% CI) | | Calibration slope (95% CI) | |
| LR | 0.0592 (0.0541, 0.0642) | | 0.1254 (0.0236, 0.2200) | | 1.0827 (0.7964, 1.3183) | |
| XGBoost | 0.0609 (0.0560, 0.0661) | | 0.0554 (-0.0523, 0.1669) | | 0.7677 (0.5568, 1.0193) | |
| LightGBM | 0.0642 (0.0615, 0.0670) | | -0.0973 (-0.1536, -0.0417) | | 1.4043 (1.0719, 1.7722) | |
| SVM | 0.0587 (0.0536, 0.0636) | | 0.0984 (-0.0064, 0.2056) | | 0.9227 (0.6748, 1.2084) | |
| GBM | 0.0613 (0.0564, 0.0665) | | 0.0656 (-0.0446, 0.1818) | | 0.8226 (0.6100, 1.0551) | |
| RF | 0.0637 (0.0598, 0.0677) | | 0.0878 (-0.0016, 0.1825) | | 0.8340 (0.5850, 1.1408) | |
| CatBoost | 0.0612 (0.0566, 0.0661) | | 0.0219 (-0.0828, 0.1303) | | 0.8911 (0.6746, 1.1396) | |
| EN | 0.0620 (0.0583, 0.0654) | | 0.1775 (0.1100, 0.2423) | | 1.5604 (1.1823, 2.0319) | |
| **Panel C.** Paired bootstrap comparisons between SVM and the other seven algorithms on the held-out internal test set. | | | | | | |
| Comparator | ΔROC-AUC (95% CI) | Holm-adjusted P (ROC) | | ΔPR-AUC (95% CI) | | Holm-adjusted P (PR) |
| LR | 0.0116 (-0.0040, 0.0275) | 1.00 | | 0.0278 (-0.0436, 0.0893) | | 0.511 |
| XGBoost | 0.0023 (-0.0284, 0.0339) | 1.00 | | 0.0776 (-0.0028, 0.1418) | | 0.180 |
| LightGBM | 0.0157 (-0.0199, 0.0516) | 1.00 | | 0.1064 (0.0207, 0.1952) | | 0.096 |
| GBM | 0.0024 (-0.0247, 0.0289) | 1.00 | | 0.0635 (-0.0137, 0.1464) | | 0.511 |
| RF | 0.0171 (-0.0216, 0.0524) | 1.00 | | 0.1507 (0.0739, 0.2186) | | 0.007 |
| CatBoost | -0.0032 (-0.0280, 0.0202) | 1.00 | | 0.0537 (-0.0163, 0.1314) | | 0.511 |
| EN | 0.0056 (-0.0090, 0.0200) | 1.00 | | 0.0428 (-0.0334, 0.1056) | | 0.511 |
| **Notes:** The training cohort included 2,155 patients (167 NOAF events). Panel A shows mean (SD) performance across two repeats of stratified 10-fold outer cross-validation (3-fold inner tuning); SDs reflect between-repeat variability, not confidence intervals. SVM was selected based on the highest mean OOF PR-AUC (bold). Panels B and C evaluate the held-out internal test set (n = 922; 71 NOAF events). Calibration was assessed after Platt scaling fitted to training-set OOF predictions. Panel C reports SVM-minus-comparator differences (positive values favoring SVM), with unadjusted 95% CIs from 1,000 outcome-stratified paired bootstrap resamples. P values were adjusted using the Holm–Bonferroni method separately for the seven ROC-AUC and seven PR-AUC comparisons. | | | | | | |
| **Abbreviations:** CatBoost, categorical boosting; CI, confidence interval; CV, cross-validation; EN, elastic net; GBM, gradient boosting machine; LightGBM, light gradient boosting machine; LR, logistic regression; NOAF, new-onset atrial fibrillation; OOF, out-of-fold; PR-AUC, area under the precision–recall curve; RF, random forest; ROC-AUC, area under the receiver operating characteristic curve; SD, standard deviation; SVM, support vector machine; XGBoost, extreme gradient boosting. | | | | | | |

| **Supplementary Table 10.** Incremental predictive performance of inflammatory indices for in-hospital NOAF after AMI. | | | | | | | | | |
| --- | --- | --- | --- | --- | --- | --- | --- | --- | --- |
| **Panel A.** Model performance in repeated cross-validation and the held-out internal test set | | | | | | | | | |
| Model | OOF ROC-AUC mean (SD) | | OOF PR-AUC mean (SD) | | Test ROC-AUC | Test PR-AUC | Test Brier | Calibration intercept | Calibration slope |
| Clinical + routine CBC (5) | 0.816 (0.003) | | 0.331 (0.017) | | 0.791 | 0.354 | 0.0605 | 0.121 | 0.913 |
| Inflammatory indices (3) | 0.648 (0.019) | | 0.117 (0.007) | | 0.645 | 0.127 | 0.0697 | −0.013 | 1.053 |
| Combined (8) | 0.836 (0.001) | | 0.356 (0.009) | | 0.798 | 0.407 | 0.0587 | 0.098 | 0.923 |
| **Panel B.** Paired test-set differences: combined model minus clinical/CBC model | | | | | | | | | |
| Metric | | | | Difference | | | Paired bootstrap 95% CI | | |
| ROC-AUC | | | | 0.0066 | | | (−0.0126, 0.0263) | | |
| PR-AUC | | | | 0.0532 | | | (0.0014, 0.1152) | | |
| Brier score | | | | −0.00185 | | | (−0.00383, −0.00005) | | |
| Calibration intercept | | | | −0.0228 | | | (−0.0626, 0.0149) | | |
| Calibration slope | | | | 0.0097 | | | (−0.0860, 0.0950) | | |
| **Panel C.** Decision-curve analysis: paired difference in net benefit (combined minus clinical/CBC) | | | | | | | | | |
| Threshold | | Δ Net benefit (pointwise 95% CI) | | | | Threshold | | Δ Net benefit (pointwise 95% CI) | |
| 2% | | −0.00308 (−0.00773, 0.00144) | | | | 12% | | 0.00030 (−0.00567, 0.00646) | |
| 3% | | 0.00003 (−0.00434, 0.00454) | | | | 13% | | −0.00054 (−0.00742, 0.00678) | |
| 4% | | −0.00014 (−0.00321, 0.00321) | | | | 14% | | 0.00093 (−0.00649, 0.00838) | |
| 5% | | −0.00211 (−0.00594, 0.00051) | | | | 15% | | −0.00006 (−0.00747, 0.00683) | |
| 6% | | 0.00282 (−0.00139, 0.00695) | | | | 16% | | −0.00124 (−0.00821, 0.00563) | |
| 7% | | 0.00323 (−0.00109, 0.00800) | | | | 17% | | −0.00200 (−0.00854, 0.00431) | |
| 8% | | 0.00099 (−0.00500, 0.00693) | | | | 18% | | −0.00214 (−0.00852, 0.00437) | |
| 9% | | 0.00206 (−0.00347, 0.00789) | | | | 19% | | −0.00153 (−0.00797, 0.00479) | |
| 10% | | 0.00229 (−0.00301, 0.00784) | | | | 20% | | −0.00380 (−0.00976, 0.00217) | |
| 11% | | 0.00080 (−0.00556, 0.00730) | | | |  | |  | |
| **Notes:** The clinical/CBC model included age, BNP, LAD, LVEF, and lymphocyte count; the inflammation-only model included MLR, NLR, and SIRI; the combined model included all eight predictors. The training cohort comprised 2,155 patients (167 NOAF events) and the internal test cohort 922 patients (71 events). All three models were developed using radial basis function support vector machines under identical resampling partitions (2 × 10-fold outer CV and 3-fold inner tuning), with OOF standard deviations calculated across two repeats. Test predictions were recalibrated using training-derived Platt scaling. Paired differences and 95% CIs were estimated using 1,000 stratified bootstrap resamples; DCA intervals were pointwise. No consistent additional net benefit was observed across 2%–20% thresholds. This post hoc ablation used predictors selected from the complete training cohort without repeating feature selection within the ablation folds. | | | | | | | | | |
| **Abbreviations:** AUC, area under the curve; BNP, B-type natriuretic peptide; CBC, complete blood count; CI, confidence interval; CV, cross-validation; DCA, decision-curve analysis; LAD, left atrial diameter; LVEF, left ventricular ejection fraction; MLR, monocyte-to-lymphocyte ratio; NLR, neutrophil-to-lymphocyte ratio; NOAF, new-onset atrial fibrillation; OOF, out-of-fold; PR, precision–recall; ROC, receiver operating characteristic; SIRI, systemic inflammation response index; RBF, radial basis function; SVM, support vector machine; AMI, acute myocardial infarction. | | | | | | | | | |

**Supplementary Figure 1:** Flow diagram for participants included in the study. Abbreviations: AF, atrial fibrillation; AFL, atrial flutter; CAG, coronary angiography; AMI, acute myocardial infarction; NOAF, new-onset atrial fibrillation.

**Supplementary Figure 2:** Multicollinearity is evaluated via the variance inflation factor (VIF) = 1/(1-R^2^).The stepwise screening process involves calculating the VIF for each variable and iteratively excluding the variable with the highest VIF if its value is ≥ 5. Abbreviations: VIF, variance inflation factors; NOAF, new-onset atrial fibrillation; BMI, body mass index; DM, diabetes mellitus; AST, aspartate aminotransferase; TG, triglyceride; HDL-C, high-density lipoprotein cholesterol; LDL-C, low-density lipoprotein cholesterol; LVEF, left ventricular ejection fraction; LAD, left atrial diameter; NLR, neutrophil-to-lymphocyte ratio; PLR, platelet-to-lymphocyte ratio; SII, systemic immune-inflammation index; MLR, monocyte-to-lymphocyte ratio; SIRI, systemic inflammation response index; AISI, aggregate index of systemic inflammation.

**Supplementary Figure 3:** Prevalence of NOAF across quartiles of six inflammatory indices. Abbreviations: NOAF, new-onset atrial fibrillation; NLR, neutrophil-to-lymphocyte ratio; PLR, platelet-to-lymphocyte ratio; SII, systemic immune-inflammation index; MLR, monocyte-to-lymphocyte ratio; SIRI, systemic inflammation response index; AISI, aggregate index of systemic inflammation.

**Supplementary Figure 4:** Subgroup analyses for the association between six inflammatory indices and the risk of NOAF. Abbreviations: NOAF, new-onset atrial fibrillation; NLR, neutrophil-to-lymphocyte ratio; PLR, platelet-to-lymphocyte ratio; SII, systemic immune-inflammation index; MLR, monocyte-to-lymphocyte ratio; SIRI, systemic inflammation response index; AISI, aggregate index of systemic inflammation.

**Supplementary Figure 5:** Individual-level interpretation of the SVM model using SHAP analysis. (A) Waterfall plot illustrating feature contributions to the predicted risk for a patient with NOAF; (B) Force plots illustrating individual feature contributions for patients with and without NOAF. Abbreviations: SVM, support vector machine; SHAP, Shapley Additive Explanations; NOAF, new-onset atrial fibrillation; LVEF, left ventricular ejection fraction; LAD, left atrial diameter; BNP, B-type natriuretic peptide; NLR, neutrophil-to-lymphocyte ratio; MLR, monocyte-to-lymphocyte ratio; SIRI, systemic inflammation response index.

**Supplementary Figure 6:** SHAP dependence plots for the eight selected predictors. Abbreviations: SHAP, Shapley Additive Explanations; LVEF, left ventricular ejection fraction; LAD, left atrial diameter; BNP, B-type natriuretic peptide; NLR, neutrophil-to-lymphocyte ratio; MLR, monocyte-to-lymphocyte ratio; SIRI, systemic inflammation response index.
